# *C9orf72* is differentially expressed in the central nervous system and myeloid cells and consistently reduced in *C9orf72*, *MAPT* and *GRN* mutation carriers

**DOI:** 10.1186/s40478-016-0306-7

**Published:** 2016-04-14

**Authors:** Patrizia Rizzu, Cornelis Blauwendraat, Sasja Heetveld, Emily M. Lynes, Melissa Castillo-Lizardo, Ashutosh Dhingra, Elwira Pyz, Markus Hobert, Matthis Synofzik, Javier Simón-Sánchez, Margherita Francescatto, Peter Heutink

**Affiliations:** German Center for Neurodegenerative Diseases (DZNE), Otfried-Müller Strasse 23, Tübingen, 72076 Germany; Department of Neurodegenerative Diseases, Hertie Institute for Clinical Brain Research, University of Tübingen, Tübingen, 72076 Germany

**Keywords:** Neurodegeneration, C9orf72 risk haplotype, Hexanucleotide repeat expansion, Frontotemporal dementia

## Abstract

A non-coding hexanucleotide repeat expansion (HRE) in *C9orf72* is a common cause of amyotrophic lateral sclerosis (ALS) and frontotemporal dementia (FTD) acting through a loss of function mechanism due to haploinsufficiency of *C9orf72* or a gain of function mediated by aggregates of bidirectionally transcribed HRE-RNAs translated into di-peptide repeat (DPR) proteins. To fully understand regulation of *C9orf72* expression we surveyed the *C9orf72* locus using Cap Analysis of Gene Expression sequence data (CAGEseq). We observed *C9orf72* was generally lowly expressed with the exception of a subset of myeloid cells, particularly CD14+ monocytes that showed up to seven fold higher expression as compared to central nervous system (CNS) and other tissues. The expression profile at the *C9orf72* locus showed a complex architecture with differential expression of the transcription start sites (TSSs) for the annotated *C9orf72* transcripts between myeloid and CNS tissues suggesting cell and/or tissue specific functions. We further detected novel TSSs in both the sense and antisense strand at the *C9orf72* locus and confirmed their existence in brain tissues and CD14+ monocytes. Interestingly, our experiments showed a consistent decrease of *C9orf72* coding transcripts not only in brain tissue and monocytes from *C9orf72*-HRE patients, but also in brains from *MAPT* and *GRN* mutation carriers together with an increase in antisense transcripts suggesting these could play a role in regulation of *C9orf72*. We found that the non-HRE related expression changes cannot be explained by promoter methylation but by the presence of the *C9orf72*-HRE risk haplotype and unknown functional interactions between *C9orf72, MAPT* and *GRN*.

**Electronic supplementary material:**

The online version of this article (doi:10.1186/s40478-016-0306-7) contains supplementary material, which is available to authorized users.

## Introduction

In 2011 two independent studies identified a non-coding HRE in the *C9orf72* gene as the major cause for chromosome 9-linked ALS and FTD with or without concomitant motor neuron disease [1, 2]. Since then, rapid progress has been made in elucidating the pathological and mechanistic aspects of the disease causing mutation. The current hypotheses suggest that the disease occurs through, not necessarily exclusive, loss- and gain of toxicity function mechanisms mediated by (1) haploinsufficiency, (2) transcription of sense and antisense HRE-RNAs and (3) translation of these RNAs into DPR proteins through unconventional repeat-associated non-ATG (RAN) translation [3, 4].

Although the exact pathogenic mechanisms are not yet fully understood, the repeat-mediated toxicity hypothesis is gaining momentum. Bidirectionally transcribed HRE containing RNAs accumulate into RNA foci, occurring mainly in the nuclei of neurons in brain tissue and cultured cells of patients [1, 5, 6]. HRE-RNA transcripts can form hairpin and G-quadruplex structures [7, 8] and induce a toxic RNA gain of function by binding and sequestering RNA-binding proteins involved in splicing [9] and nucleocytoplasmic trafficking [10, 11] consequently altering their availability for their normal function.

The C9orf72 DPR proteins accumulate into cytoplasmic and intranuclear inclusions in brains of patients [12–16]. And studies in cell culture and animal models strongly corroborate that overexpression of DPR proteins is toxic and can induce nuclear inclusions and nucleolar stress [17, 18].

A loss of function mechanism for *C9orf72* has also been suggested, based on the observed decrease in *C9orf72* mRNA expression in brain tissue and iPSC derived-neurons of *C9orf72*-HRE patients. This reduction in expression could be mediated by the G-quadruplex structures formed by the HRE-RNAs, which would impair *C9orf72* full-length transcription as a result of the repeat-length dependent accumulation of aborted transcripts of *C9orf72* [17].

Further evidence for this hypothesis comes from the targeted reduction of the *C9orf72* orthologue in zebrafish that resulted in axonopathy and motor deficits and from a *C.elegans C9orf72* knockout model that presented with motor phenotypes, suggesting that loss of C9orf72 protein can lead to motor deficits [19, 20]. However, silencing of *C9orf72* by intracerebroventricular delivery of antisense oligonucleotides in adult mice or by neural-specific ablation in conditional *C9orf72* knock-out mice [5, 21] did not lead to motor or behavioral phenotype arguing against a loss of function as the primary pathogenic mechanism. But even though *C9orf72* reduction might not be the major culprit, it could still be detrimental to cells as substantial evidence supports *C9orf72* interrelated roles in protein trafficking [22–24] and autophagy [25]. In this context and considering possible therapeutic approaches, it becomes important to fully understand how *C9orf72* RNA expression is regulated. Up until now, the attention of the field has been mainly focused on the repeat expansion and the immediate neighboring sequence, with several studies suggesting that epigenetic changes like the *C9orf72* promoter hypermethylation, might partially contribute to transcriptional silencing of mutant *C9orf72* [26, 27]. To help understand additional mechanisms contributing to *C9orf72* regulation and *C9orf72* loss of function we sought to characterize the transcriptional landscape of the *C9orf72* locus taking a broader approach. By surveying the global CAGEseq expression data generated by single-molecule cDNA sequencing in the context of the FANTOM5 project [28] we observed that transcription at the *C9orf72* locus has a complex architecture.

We found that the TSSs for the annotated *C9orf72* transcripts are remarkably differentially expressed across samples, particularly between a subset of myeloid cells and CNS tissues. We detected novel non-annotated TSSs on the sense and antisense strand at the *C9orf72* locus suggesting new potential transcripts and we observed changes in the expression of the annotated and newly identified *C9orf72* transcripts, not only in *C9orf72*-HRE patients, but also in FTD patients carrying mutations in the microtubule associated protein tau (*MAPT*) and granulin (*GRN*) genes. We demonstrate that the repeat expansion cannot fully explain the reduction in *C9orf72* expression and that additional molecular mechanisms contribute to the regulation of *C9orf72* expression.

## Material and methods

### CAGEseq datasets

- Dataset 1: CAGEseq data published in the context of the FANTOM5 promoterome project [28], which generated a comprehensive map of TSSs and their usage across 975 human and 399 mouse samples (the samples used in this work to generate the individual and combined TSSs expression profiles of CNS and myeloid cells are listed in Additional file 1: Table S1).
- Dataset 2: CAGEseq data from 144 human brain samples consisting of seven anatomical regions (occipital-, frontal-, temporal lobe, hippocampus, caudate, putamen and cerebellum) from seven controls and three groups of familial FTD patients carrying a *C9orf72-HRE*, *MAPT* or *GRN* pathogenic mutation (list of samples provided in Additional file 1: Table S2).
- Dataset 3: CAGEseq data from human frontal lobe of 119 control donors with ages ranging from 2 to 95 years presenting no neurological symptoms at time of death (C. Blauwendraat, under review in another journal).

### Definition of distinct tag clusters at the human *C9orf72* locus using FANTOM5 CAGEseq data

From the ZENBU genome browser [29] we downloaded the coordinates of the decomposition-based peak identification (DPI) clusters identified by the FANTOM5 project in the genomic region chr9:27572518-27574055 (human genome build GRCh37/hg19), which includes the 5′ end of the *C9orf72* gene and encompasses all the CAGEseq expression signals for the *C9orf72* locus. Each DPI cluster, composed by multiple adjacent CAGEseq reads identifies a TSS. A detailed description of the DPI clustering method can be found in the supplementary information of [28] section 4, along with the definition of robust and permissive DPIs.

Fifteen TSSs have been identified in the region, four on the forward and 11 on the reverse strand (Additional file 1: Figure S1A-B). This original list of TSSs was adjusted based on the actual CAGEseq expression profiles (Additional file 1: Figure S1A) and resulted in the definition of a final set of nine TSSs, three on the forward and six on the reverse strand (Additional file 1: Figure S1C). TSSs on the reverse strand (same transcriptional direction as *C9orf72)* were named consistently with *C9orf72* transcript variants when possible, with letters to separate distinct TSSs associated to the same variant (S4 indicates a putative novel transcript). TSSs on the forward strand were named AS (to reflect the fact that they are antisense to *C9orf72*) and numbered in the 5′ to 3′ direction.

Manual modifications to the original list of TSSs (Additional file 1: Figure S1D): we merged neighboring DPI clusters with similar expression profiles (giving rise to S4, AS1 and S2C TSSs), we extended two DPI clusters in order to capture all the CAGEseq signals corresponding to them (giving rise to S1 + S3a and AS3 TSSs) and we added two DPI clusters suggested by the permissive set of DPI clusters (TSSs AS2 and S1 + S3b). The remaining TSSs (S2B and S2A) were simply renamed and maintain the same coordinates as the original DPI clusters.

### Expression of *C9orf72* TSSs as defined by CAGEseq using dataset 1

For each of the TSSs defined as described above, we downloaded from ZENBU tag-count per million (tpm) normalized expression values for all the samples available in the FANTOM5 promoterome study. The downloaded expression values for CNS and myeloid cells are provided in Additional file 1: Table S1.

### Expression of *C9orf72* TSSs as defined by CAGEseq using dataset 2

CAGEseq libraries were prepared using a published protocol [30]. Briefly, total RNA from 144 brain samples (Additional file 1: Table S2) was used as starting material and sequenced on a HiSeq 2000 (Illumina). Sequenced reads were demultiplexed, trimmed using the FASTX toolkit (FASTX toolkit, http://hannonlab.cshl.edu/fastx_toolkit/), filtered for artifacts using TagDust (version 1.12) [31] and mapped to the human genome (build GRCh37/hg19) using the Burrows-Wheeler Aligner (BWA version 0.5.9) [32] allowing up to two mismatches. The resulting BAM files were uploaded in ZENBU and the tpm normalized expression values of the *C9orf72* TSSs were downloaded (available in Additional file 1: Table S2).

### Expression of *C9orf72* TSSs as defined by CAGEseq using dataset 3

Library preparation and creation of the BAM files containing mapped reads was performed as described above. The mapped CAGEseq reads were grouped into single base pair promoters by determining all positions in the genome to which the 5′ end of at least one CAGE read with mapping quality of at least 20 mapped to and outputting tpm normalized expression values per base pair position [33].

### *C9orf72* expression in aging brain

We analyzed the CAGEseq dataset 3 obtained from 119 frontal cortex brain samples to assess whether brain *C9orf72* expression is influenced by age.

Pearson correlation calculations were made between each newly defined *C9orf72* TSS and age value. To identify possible hidden confounding effects we also determined whether any of the *C9orf72* TSS was influenced by RNA integrity number (RIN), gender and postmortem interval (pmi).

### Weighted Gene Coexpression Network Analysis (WGCNA)

We downloaded from the FANTOM5 data repository the BAM files corresponding to eosinophils, neutrophils, CD14+ monocytes and all adult CNS samples (the full list of samples included in the analysis is provided in Additional file 1: Table S1). After removing reads mapping to chromosomes M and Y from the downloaded BAM files, we used Python scripts designed at the RIKEN Omics Science Center [33] to group the remaining CAGEseq reads into TSSs and obtain the corresponding tpm normalized expression values. Because the major aim of the analysis was to infer information about *C9orf72* function, we decided to include in the WGCNA only TSSs mapping to annotated genes. The data were filtered to ensure that all TSSs had expression above 1tpm in at least one sample and log-transformed, resulting in a dataset including 26,305 TSSs mapping to 17,267 distinct genes. Co-expression network construction, (including module membership calculation) was performed as previously described [34] and resulted in 18 co-expression modules, three of which contained all the TSSs mapping to *C9orf72* (turquoise, yellow, blue). Module membership measure for each *C9orf72* TSSs was also calculated. Of these only the S1 + S3b TSS showed higher membership with respect to a different module (pink module). For the modules turquoise, yellow, blue and pink we calculated correlation values between the expression of *C9orf72* TSSs and all the other TSSs assigned to the module. The correlations were calculated using the R function cor.test() with “spearman” method option, resulting in an association test based on Spearman’s rho statistic. Adjusted *p*-values were calculated using the R function p.adjust(), with method = “BH” option (Benjamini-Hochberg adjustment designed to control FDR). Networks and functional analysis were generated through the use of Qiagen’s Ingenuity Pathways Analysis (IPA, QIAGEN Redwood City).

### Donor samples used in quantitative PCR (qPCR) experiments

Frozen tissue from disease and control donors was obtained from the Netherlands Brain Bank for the following brain regions: medial frontal and temporal gyrus, superior occipital gyrus, cerebellum, hippocampus, caudate and putamen. We obtained tissues from sporadic-FTD patients and patients with *C9orf72*-HRE, *MAPT* and *GRN* mutations, sporadic cases with Alzheimer’s disease (AD), Progressive Supranuclear Palsy (PSP), ALS, Multiple Sclerosis (MS), Parkinson’s disease (PD), cases with Huntington’s disease (HD) and non-demented control donors.

Peripheral blood was collected from healthy donors and patients with clinical diagnosis of non familiar FTD, ALS and PD. Following appropriate informed consent genomic DNA, RNA and CD14+ monocytes were isolated for further analysis (see below). Details about brain samples are listed in Table 1 and details on blood samples are provided in Table 2.

**Table 1:** Characteristics of investigated brain samples

Disease code	Sample number	Age	Female frequency	Risk haplotype		pmi	RIN
				Hom	Het		
NDD	9	80.3 ± 10.7	4 (50 %)	1	4	5:46 ± 1:27	8.4 ± 0,8
C9orf72-HRE	10	64.9 ± 9.0	7 (70.%)	1	9	6:18 ± 1:25	7.4 ± 1.2
FTD-MAPT	16	62.13 ± 8.05	8 (50 %)	2	7	6:11 ± 1:50	7 ± 1.1
FTD-GRN	8	61.6 ± 8.1	5 (62.5 %)	0	2	4:49 ± 1:00	6.9 ± 0,9
AD	5	82 ± 5.04	3 (60 %)	0	1	4:31 ± 1:18	7.2 ± 1,5
PSP	5	71.6 ± 9.	3 (60 %)	0	5	6:47 ± 1:46	7 ± 1.3
FTD sporadic	4	60.3 ± 10.8	2 (50 %)	1	NP	6:02 ± 1:18	7.3 ± 1.5
ALS	3	57,3 ± 21.1	1 (33.3 %)	0	1	5:28 ± 0:27	9 ± 0.05
HD	3	53.3 ± 6.6	1 (33.3 %)	0	2	7:16 ± 2:43	4.9 ± 0.6
MS	3	64.3 ± 21.5	2 (66.7 %)	0	2	6:06 ± 2:48	7.9 ± 1.3
PD	3	79.7 ± 8	0	0	1	4:55 ± 1:07	7.9 ± 0.9
Total	69			5 (7.24 %)	34 (49.3 %)		

The table describes characteristics of brain samples used in this study: age, gender, sharing of risk haplotype, post mortem interval (pmi) and RNA integrity number (RIN). *NDD* non-demented donors, Age, pmi and RIN: per disease group indicated average and standard deviation

FTD-*MAPT* cases carrying pathogenic mutations: ten cases carrying the P301L mutation; four cases with the G272V mutation; one case with the R406W mutation and one case with the L315R mutation. FTD-*GRN* cases carrying pathogenic mutations: five cases carrying the S82Vfs mutation; one case with G300X mutation; one case with the C105fs mutation and one case carrying the Q24X mutation

**Table 2:** Characteristics of the investigated CD14+ donor samples

Disease code	Sample number	Age	Age at onset	Female frequency	Risk haplotype	
					Hom	Het
Controls	5	35.2 ± 13.3	NA	40 %	NT	NT
C9orf72-HRE	4	62.5 ± 9.9	58 ± 12.3	50 %	2	2
ALS	9	65.9 ± 6.8	62.8 ± 6.5	44.4 %	0	1
FTD	9	63 ± 13.2	60.3 ± 13.3	33.3 %	0	3
PD	10	66.8 ± 9.4	56.5 ± 7.9	30 %	0	5

The table summarizes characteristics (age, age at onset, gender, sharing of risk haplotype) of CD14+ sample donors

*NA* not applicable, *NT* non-tested. Age and age at onset: per group indicated average and standard deviation

All samples used in this study were screened for the presence of the *C9orf72-*HRE by primed repeat PCR according to established protocols [1, 2]. Reported mutations in *MAPT* and *GRN* mutations carriers were sequence verified. Brain donors with the diagnosis of sporadic FTD were also screened for mutations in the *MAPT* and *GRN* genes. Additionally we amplified 1.6 kb in the *C9orf72* promoter region in *MAPT* and *GRN* mutations carriers. PCR products were purified with EXO-SAP (GE Healthcare) and sequenced by using BigDye terminators v3 on an ABI3500 system (Thermo Fisher Scientific). Primers sequences are supplied in Additional file 1: Table S3.

### DNA and RNA isolation

Genomic DNA was isolated from peripheral blood and frozen brain tissue by using the DNeasy blood and tissue kit (Qiagen) following the manufacturer protocol.

RNA was isolated from frozen brain tissue, CD14+ monocytes and human microglia cells derived from fetal brain tissue purchased from 3H Biomedical with TRIzol reagent (Thermo Fisher Scientific) followed by DNAse treatment and further purification with the RNeasy columns (Qiagen). RIN was determined on a Bioanalyzer 2100 system (Agilent Technologies Inc.) (Table 1).

### CD14+ monocytes isolation

CD14+ monocytes were isolated from peripheral blood mononuclear cells by density gradient using Ficoll-Paque™ (GE Healthcare), followed by labeling with CD14 microbeads and magnetic separation with LS columns using the protocol provided by Miltenyi Biotech. CD14+ monocytes isolation with >90 % purity was verified by cell analysis on a FACSCalibur cytometer (BD Biosciences) using CellQuest software with gating on a live cell forward/side scatter gate, using the CD14 PE-conjugated and Mouse IgG2a-PE conjugated isotope control antibodies (Miltenyi Biotec) according to the protocol provided by the manufacturer.

### cDNA synthesis, strand specific reverse transcriptase PCR, 3’RACE and qPCR

Total RNA primed with oligo dT (Qiagen) and random decamers (Thermo Fisher Scientific) was used for cDNA synthesis with Superscript III reverse transcriptase (RT) (Thermo Fisher Scientific) according to manufacturer’s specifications. Possible DNA genomic contamination was excluded by adding RT(-) reactions.

Strand specific RT- PCR (ssRT-PCR) was performed according to Chung et al [35].

RACE ready mRNA for 3’RACE experiments were performed using the SMARTer RACE cDNA amplification kit (Clontech Laboratories) according to the manufacturer protocol. Primers sequences are given in Additional file 1: Table S3.

qPCR was carried out in triplicate on a ViiA7 real time PCR system (Thermo Fisher Scientific) using SYBR Green PCR master mix (Thermo Fisher Scientific) and 0,04 μM specific primer pairs for all targets. For a subset of samples we performed qPCR with Taqman assays for total *C9orf72* and transcript 3 in triplicate using Taqman Gene expression Master mix (Thermo Fisher Scientific) according to the procedure recommended by the manufacturer.

Normalized relative quantities (NRQ) were calculated using qbase^+^ version 2.4 (Biogazelle) with *B2M, HMBS, RPLPO* and *OAZ1* as reference targets [36]. The geNorm module in qbase^+^ was used to compute expression stability values for all reference targets and determine the optimal number of reference targets for every experiment. Variables were compared using nonparametric Mann-Whitney tests and a *p*-value of 0.05 was considered significant.

To measure absolute *C9orf72* quantity in brain and monocytes cDNAs, we performed digital PCR using the Quant studio 3D system (Life Technologies) using cDNA serial dilution (25 ng-3.125 ng RNA range) and Taqman assays for *C9orf72* total and transcript 3 according to the manufacturer ‘s protocol.

Primers for *C9orf72* total, transcript 1,2 and 3 were obtained from Waite et al. [37]. Primer sequences for S4-TSS, AS1 and AS3 are listed on Additional file 1: Table S3.

### *C9orf72* expression quantitative trait loci analysis using CAGEseq expression values

Genome wide genotypes data for 119 frontal lobe samples for which we have CAGEseq expression values were obtained as described in Blauwendraat et al (C. Blauwendraat under review in another journal).

We selected 20 Single Nucleotide Polymorphisms (SNPs) defining the *C9orf72-HRE* risk haplotypes as described by Mok et al [38] and haplotypes were constructed based on the reported risk allele allowing maximum two mismatches per individual. Expression quantitative trait loci (eQTL) analysis was performed using MatrixeQTL [39] testing the expression level of the newly defined TSSs with the frequency of risk haplotypes per individual, using as covariates pmi, age, gender and RIN.

### *C9orf72* eQTL analysis using normalized relative quantities expression values

Normalized relative quantities (NRQ) expression values were generated in qPCR experiments for the different *C9orf72* targets as described above.

Five SNPs were selected for genotyping based on the reported risk allele and their genomic location: rs10757665, rs10757668, rs3849942, rs2453556, and rs702231. Haplotypes were constructed to identify carriers of the *C9orf72-HRE* risk allele allowing maximum two mismatches per individual at the ends of the haplotype as previously described [38]. eQTL analysis was performed using MatrixeQTL correlating the NRQ expression levels of the qPCR targets with the frequency of risk haplotypes per individual using as covariates brain region, disease status and disease mutation.

### *C9orf72* promoter methylation assay

Quantitative assessment of methylation levels was determined by using a methylation-sensitive restriction enzyme DNA digestion coupled with qPCR [40].

We applied Mann-Whitney U tests to compare methylation levels between *C9orf72*–HRE patients and controls, *MAPT* mutation carriers and controls and *GRN* mutations carriers and controls. Pearson correlation analysis was performed to correlate methylation values with risk haplotype frequency and expression.

### Knock down experiments

Knock down experiments were performed on BE(2)M17 cell lines (ATCC® CRL-2267™) using short hairpin RNA (shRNA) plasmids for *C9orf72* transcripts, 2 and 3, *MAPT, GRN* and scrambled control (Sigma, TRC 1 and 1.5) as described in [41].

### RNA FISH and analysis of RNA foci burden

CD14+ monocytes from three *C9orf72*-HRE patients and three FTD cases HRE negative were seeded onto Poly-L-lysine-coated glass coverslips by cytospin at 120 g for 5 min, fixed in 4 % paraformaldehyde and stained with 5’ TYE563-labelled [CCCGG]_3_ probe (sense foci), or [GGGGCC]_3_ probe (antisense foci) (Exiqon) as previously described [5]. Nuclei were stained with Hoechst and slides were subsequently imaged on a Leica TCS SP8 confocal microscope using a Leica Plan Apochromat 100× oil immersion objective lens. The resulting images were analyzed using an internally developed pipeline in CellProfiler [42]. Briefly, maximum projections of each image channel were made in Fiji [43] and used as input in the pipeline for at least 70 cells for each patient and probe. Threshold value was automatically set for each image and nuclei were detected. Speckles were then enhanced in the RNA foci channel, and this channel was subsequently masked based on the nuclei channel. Threshold was set per object basis using the Mixture of Gaussian algorithm. The RNA foci were then automatically counted and assigned to a nucleus, and the percentage of nuclei containing at least one focus was determined for each sample.

### Statistical analysis and plots

All statistical analyses, correlations and plots were performed using the free software environment R (Environment R, (https://www.r-project.org/).

## Ethical approval and consent to participate

Frozen tissues used to generate the CAGEseq dataset 2 and used in qPCR studies was obtained from the Netherlands Brain Bank (NBB). All tissue requests received at the NBB are reviewed by NBB's scientific committee and all material and data collected by the NBB are obtained on the basis of written informed consent. Procedures, information and consent forms of the NBB have been approved by the Medical Ethics Committee of the VU Medical Centre. The use of human brain samples for the work leading to the generation of the CAGEseq dataset 3 was approved by the NIH Office for Human Subjects Research as indicated in C. Blauwendraat, under review in another journal. Controls and disease blood donors gave informal consent and study approval was obtained by the Ethics Committee of the University of Tübingen.

## Results

### The *C9orf72* locus shows a complex transcriptional architecture in the CNS and in CD14+ monocytes

**Fig. 1:**
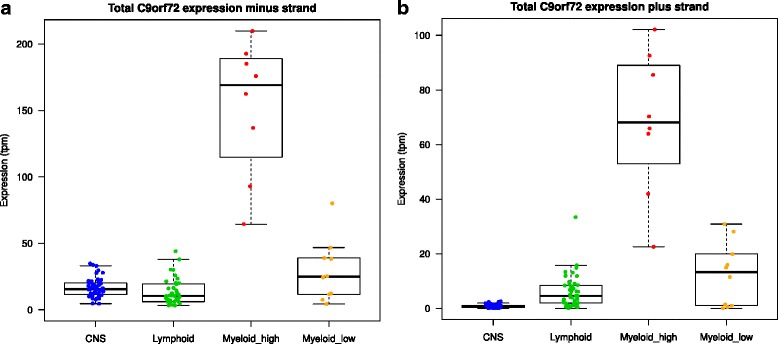
Global analysis of *C9orf72* expression using CAGEseq expression data from the FANTOM5 project. Boxplots showing total *C9orf72* expression at the *C9orf72* locus from the sense (**a**) and antisense (**b**) strand in myeloid, lymphoid and CNS samples available in the FANTOM5 promoterome sample collection. *C9orf72* expression is particularly high in a subset of myeloid cells (CD14+ monocytes, eosinophils and neutrophils) indicated as myeloid-high and lower in macrophages and mast cells (myeloid-low), T and B cells (lymphoid cells) and tissues from the CNS. Expression values are indicated on the Y-axes as tpm. Total expression at *C9orf72* locus deriving from the sense strand (reverse or -) was calculated by adding the expression of all the TSSs on the reverse strand (i.e. S1 + S3b, S1 + S3a, S2A, S2B, S2C and S4) for all the libraries included in the study. Similarly total expression deriving from the antisense strand (forward or +) was calculated adding the expression of all the TSSs located on the forward strand (i.e. AS1, AS2 and AS3)

To characterize the transcriptional events occurring at the *C9orf72* locus and identify possible new transcripts on the sense and antisense strands that contribute to *C9orf72* expression, we used publically available CAGEseq expression data generated in the context of the FANTOM5 project [28] to investigate in which tissues and cells *C9orf72* is expressed. We observed *C9orf72* was expressed above 1 tpm in the sense strand in 83.3 % of the samples and in the antisense strand in 22.1 % of the samples. Particularly, *C9orf72* expression was very high in CD14+ monocytes, eosinophils, and neutrophils, a subset of myeloid cells involved in innate and adaptive immunity, from now on referred to as myeloid-high (Fig. 1a and b). *C9orf72* expression levels in other myeloid cells such as mast cells and macrophages that are derived from monocytes (further referred to as myeloid-low), or lymphoid-derived cells like T and B cells were much lower (Fig. 1a and b). In the remaining cell types and tissue samples throughout the body including the CNS, *C9orf72* expression levels were generally low and comparable to lymphoid cells.

Interestingly we observed an increase in *C9orf72* expression from both strands after challenging CD14+ monocytes with a range of pathogens including *Candida*, *Cryptococcus*, *Streptococcus* group A and *Salmonella* that induce an immune response (Additional file 1: Figure S2). Taken together these findings suggest *C9orf72* might play a role related to myeloid function in immune response and the maturation of monocytes into macrophages.

**Fig. 2:**
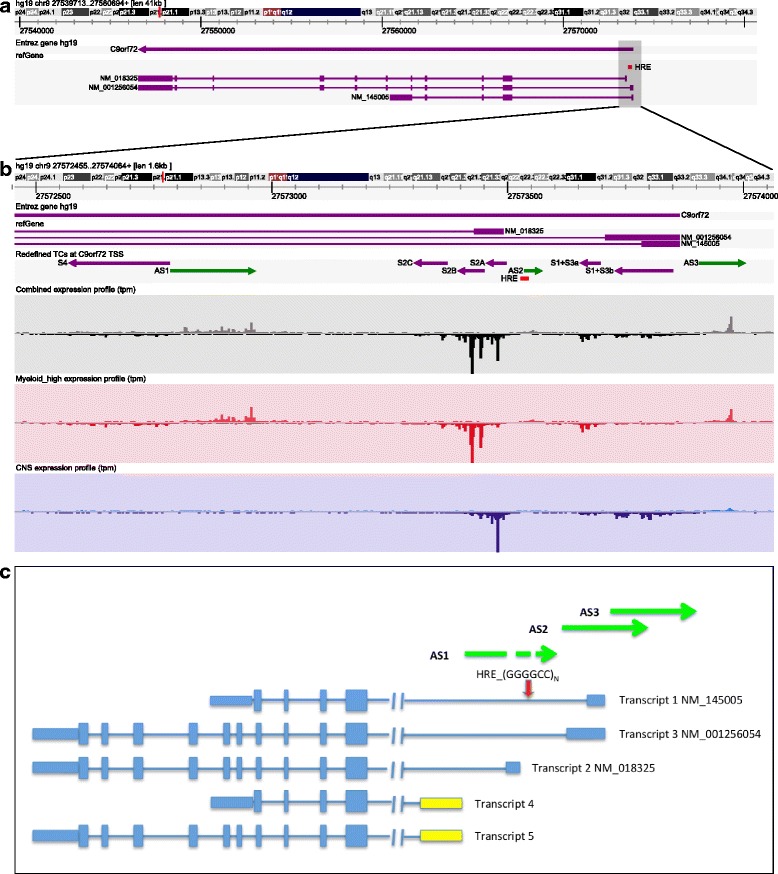
*C9orf72* locus. **a** Represents the three coding transcripts for the *C9orf72* gene located on the reverse strand of chromosome 9: NM_145005 (transcript 1), NM_18325 (transcript 2) and NM_1256054 (transcript 3). The HRE is located either in intron 1 considering transcripts 1 and 3, or at the promoter region considering transcript 2. The region captured in the figure encompasses the 41 kb identified by the hg19 coordinates chr9:27539713-27580694. **b** Zooming in of the 1,6 kb region identified by the hg19 coordinates chr9:27572455-27574064 (*grey box* in **a**) and depicting the 5′ end of the *C9orf72* locus as represented by the ZENBU Genome Browser. As indicated by the labels in the left side of each track, the following features are depicted from top to bottom: Entrez gene; refGene transcripts; *C9orf72* TSSs (the details about their definition is available in material and methods); the CAGEseq expression profile obtained combining myeloid, lymphoid and CNS samples available in the FANTOM5 tissue collection; the expression profile restricted to myeloid-high samples and the expression profile restricted to CNS samples. The height of the expression profiles is scaled to the maximum value for the corresponding set of samples and it clearly highlights the differences between TSS usage in CNS and myeloid-high samples. The actual expression levels are represented as tpm in Additional file 1: Figure S3. **c** Schematic representation of the *C9orf72* locus showing the three AS transcripts *C9orf72-AS1, C9orf72-AS2* and *C9orf72-AS3*, *C9orf72* annotated transcripts 1, 2 and 3 and the newly identified *C9orf72* transcripts 4 and 5. The dash-arrow *in C9orf72*-AS1 indicates the transcript might be longer than what detected in our experimental set-up. *C9orf72*-AS is located at chr9: 27573532-27574512 (hg19) immediately adjacent to the *C9orf72*-HRE. *C9orf72*-AS3 chr9: 27573906-27575066 (hg19) starts 40 bp downstream exon 1a of *C9orf72* presents a conserved poly-adenylation (poly-A) 692 bp from the 5′end and an alternative poly-A site 1029 bp from the 5′ end

We then analyzed TSSs expression profiles at the *C9orf72* locus, from the myeloid-high and CNS samples, in more detail. Three coding transcripts are currently annotated for the *C9orf72* gene: NM_145005 (Transcript 1), NM_18325 (Transcript 2) and NM_1256054 (Transcript 3) (Fig. 2a). The *C9orf72*-HRE is located in intron 1 considering transcripts 1 and 3, or at the promoter region considering transcript 2. Our combined TSSs profile (Fig. 2b) shows that transcripts 1 and 3 share two TSSs referred to as S1 + S3a and S1 + S3b. The S1 + S3a TSS is mainly expressed in myeloid-high cells, while the S1 + S3b TSS is mainly used in the CNS (Fig. 2b and Additional file 1: Figure S3). Transcript 2 uses three TSSs: S2A, S2B and S2C. The S2A TSS is expressed at comparable levels in CNS and myeloid cells (similar average expression as indicated in Additional file 1: Figure S3), while S2B and S2C TSSs are mainly expressed in myeloid cells (Fig. 2b and Additional file 1: Figure S3).

We observed additional TSSs, expressed mainly in myeloid cells that indicate potential novel transcripts at the *C9orf72* locus: a TSS on the reverse strand downstream of exons 1A and 1B of the annotated transcripts (S4 TSS in Fig. 2b) and three TSSs on the forward strand (AS1, AS2 and AS3 TSSs in Fig. 2b)*.* By performing a series of RT-PCR and ssRT-PCR experiments in combination with 3’ RACE from RNA of CD14+ monocytes we confirmed the existence of five new transcripts at the *C9orf72* locus in *C9orf72*-HRE patients and controls, two additional *C9orf72* transcripts and three transcripts antisense to *C9orf72* (Fig. 2c and Additional file 1: Figure S4). The novel *C9orf72* sense transcripts differ from the annotated ones only by an alternative exon 1 downstream the HRE (Fig. 2c), 265 bp long with a conserved donor splice site (AAG/gtacgt). Usage of the alternative exon 1 does not yield additional translational start sites and therefore does not change the protein composition. The three antisense *C9orf72* transcripts AS1, AS2 and AS3 (Fig. 2c) are unspliced. CAGEseq data and 3′RACE experiments show that AS1, located in intron 1b starts 657 base pairs upstream of the HRE but it does not encompass the HRE region. While we cannot rule out a technical artifact in our experimental set up because of the difficulty to reverse transcribe the HRE region, our findings are in agreement with a previous study that showed antisense transcripts starting in intron 1b, (251–455 bp upstream the repeat) but not extending to the sense exon 1a region adjacent the HRE [12]. The *C9orf72*-AS2 and -AS3 transcripts overlap for 606 bp and they are located head-to-head to *C9orf72* downstream the HRE (Fig. 2c).

The distinct modes of expression of *C9orf72* TSSs in CNS and myeloid cells are mirrored at a functional level based on the WGCNA performed on CNS, monocytes, eosinophils and neutrophils samples (Additional file 1: Table S4, and Additional file 1). Our functional enrichment analysis showed that the S2A expression profile correlates with genes involved in brain-related biological processes like synaptic transmission and vesicle transport, while the TSSs that are mainly expressed in myeloid cells are associated with immune response (Additional file 1: Table S5). Interestingly, the S1 + S3b TSS for transcript 1 and 3 is significantly correlated (rho = 0.49, adjusted *p*-value = 0,) with the *IPO7* gene encoding for importin 7, a nucleocytoplasmic transport protein [44] when considering correlations in the pink module, with which the S1 + S3b TSS showed highest membership (Additional file 1).

### *C9orf72* expression levels in CNS do not change during development and aging

**Fig. 3:**
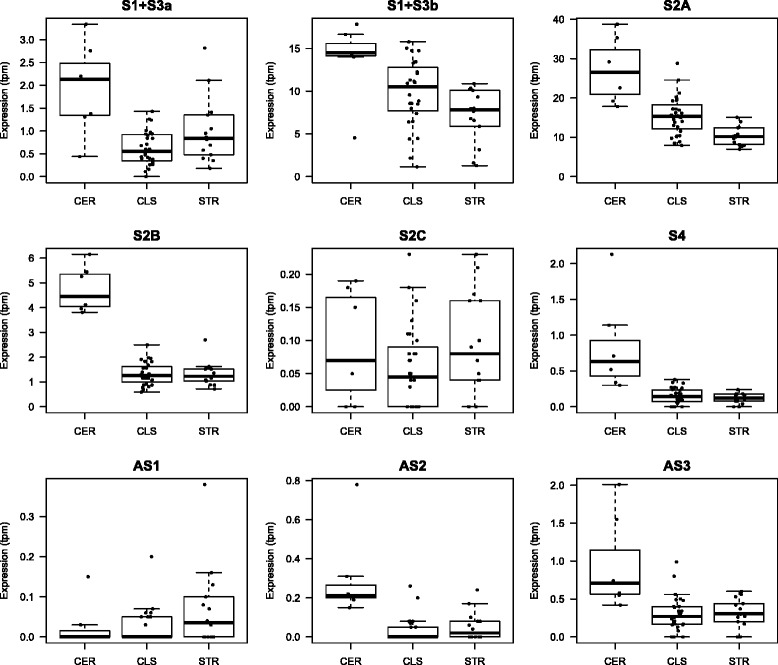
Distinct *C9orf72* TSSs expression values in the CNS of control donors. Boxplots showing expression values in tpm on the Y-axes for each TSS at the *C9orf72* locus from analysis of 49 CAGEseq libraries prepared from seven brain regions from seven control donors. On the X-axes are the CNS regions for which we present the TSSs expression values: cerebellum (CER), cortex tissues (occipital, frontal and temporal) and hippocampus (CLS), caudate and putamen (STR). S1 + S3b and S2A TSSs show the highest expression values

Since human FANTOM5 data showed considerable differences in *C9orf72* expression between myeloid cells and CNS, we investigated whether this finding was also true across distinct CNS regions. In addition to the FANTOM5 collection that contains data for a range of CNS regions in a limited numbers of adult donors, we analyzed an additional CAGEseq brain dataset consisting of 49 control libraries generated in our laboratory, with data from seven CNS regions from seven control donors (Additional file 1: Table S2). In both datasets all the TSSs for the sense transcripts showed the highest expression in cerebellum, followed by regions belonging to the cortex-limbic group and striatum (Fig. 3 and Additional file 1: Figure S5). Expression levels of the TSSs for the antisense transcripts were generally low or absent in specific CNS regions. Only AS3 TSS displayed expression above zero in all the CNS regions.

We next investigated *C9orf72* expression differences between fetal and adult cortex. The expression of *C9orf72* TSSs was generally similar in fetal and adult samples (Additional file 1: Figure S6) suggesting *C9orf72* expression levels do not significantly change during development and aging, which is in agreement with previous studies based on microarrays [45]. To confirm this result we analyzed an additional CAGEseq dataset of 119 frontal cortex brain samples with age range from 2 to 95 years from control donors generated in our laboratory. Pearson correlations between each newly defined *C9orf72* TSS and age value showed no evidence that aging influences *C9orf72* expression (data not shown).

The human FANTOM5 collection does not contain data on *C9orf72* expression in microglia. Therefore we isolated RNA from commercially available microglia from human fetal brain tissue. Digital PCR experiments (Additional file 1: Figure S7) showed *C9orf72* is lowly expressed in microglia as compared to CD14+ monocytes or total brain tissue. This finding would need however to be further investigated in adult microglia.

### *C9orf72* expression levels are reduced in FTD brains

It has been shown that the *C9orf72*-HRE generates truncated transcripts containing the repeat sequences [17, 46] therefore we wanted to determine whether the HRE sequence itself in addition harbors a TSS that could generate additional HRE-containing transcripts. CAGEseq is a technique particularly suitable for detecting TSSs of 5′-capped transcripts generated by RNA polymerase II. Analysis of our CAGEseq libraries showed no evidence for the presence of a TSS within the repeat sequence suggesting that there is either no transcription starting within the repeat or, alternatively, there is a TSS within the repeat but the corresponding transcript that would contain the expanded repeat is not capped and therefore cannot be detected with CAGEseq.

**Fig. 4:**
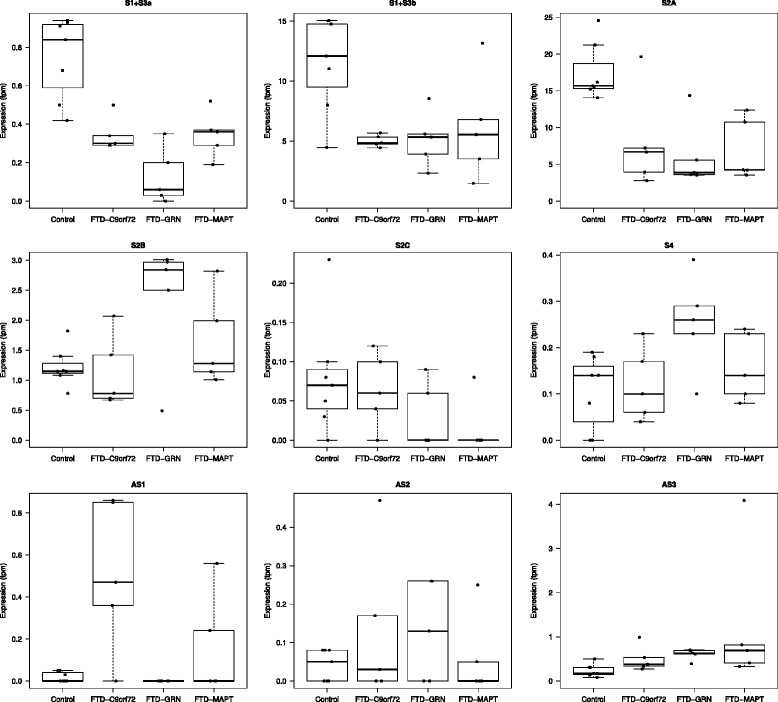
Comparison of distinct *C9orf72* TSSs expression values in frontal cortex of controls and FTD cases. Boxplots showing TSSs expression values in frontal cortex (indicated in tpm on the Y-axes) of seven controls, 5 FTD-*C9orf72*-HRE carriers, 5 FTD-*GRN* and 5 FTD-*MAPT* mutation carriers. Stark reduction for the highly expressed TSSs in CNS (S2A and S1 + S3b) is observed in all cases as compared to controls while there is an increase of AS1 TSS in *C9orf72*-HRE cases and of AS3 TSS in all FTD cases as compared to controls

Next we investigated whether the presence of the HRE would influence the expression levels of the surrounding TSSs at the *C9orf72* locus in brain tissue of *C9orf72*-HRE patients as compared to controls. Several reports have indeed observed a decrease of *C9orf72* expression in brains of *C9orf72*-HRE carriers albeit with some inconsistencies [1, 13, 19, 37, 46–50]; however these studies investigated the expression level for the three *C9orf72* annotated transcripts only. We looked at our CAGEseq data obtained from brain tissue of FTD patients carrying the *C9orf72* repeat expansion and controls. In all the investigated regions we observed a consistent reduction in expression for S1 + S3a, S1 + S3b and S2A TSSs in *C9orf72-*HRE cases as compared to control brains. In contrast we found a small increase in AS1 TSS in all brain regions and a small increase in AS3 TSS in frontal and hippocampus in cases compared to the controls (Fig. 4 and data not shown).

A recent study observed a *C9orf72* reduction in FTD patients without a *C9orf72*-HRE but this observation was not investigated in detail [19]. We therefore expanded our study to include FTD cases with *MAPT* and *GRN* mutations without the HRE. In these cases we observed decreased expression for S1 + S3a, S1 + S3b and S2A TSSs in all the investigated areas. AS3 TSS showed to be slightly increased in frontal lobe and hippocampus, while no differences where observed in AS1 TSS expression (Fig. 4 and data not shown).

To confirm and extend these findings we investigated *C9orf72* expression of all transcripts by qPCR experiments. We used a set of primers designed to target the annotated and the novel transcripts variants identified in this study in the sense and antisense orientation in a group of patients carrying *C9orf72*-HRE, *MAPT* and *GRN* mutations and control donors across several brain regions (see Table 1).

**Fig. 5:**
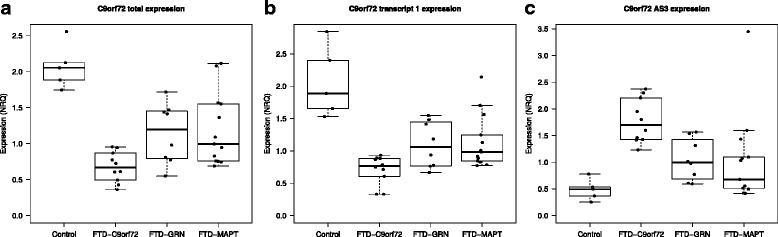
*C9orf72* reduction and *C9orf72-AS* increase in FTD brains. Quantitative comparisons of *C9orf72* expression in frontal cortex of controls and FTD brains: reduction in the expression of *C9orf72* total (**a**) and transcript 1 (**b**) is shown in *C9orf72*-HRE, FTD-*GRN* and FTD-*MAPT* mutation carriers. **c** Increase of *C9orf72-AS3* in all FTD cases as compared to controls. Expression values are expressed as NRQs values, measurements are performed in triplicates and error bars reflect standard deviations

Consistent with our CAGEseq findings we observed reduction of all *C9orf72* annotated transcripts in the *C9orf72* patients as well as in the *GRN* and *MAPT* mutation carriers in particular in frontal and temporal cortex and hippocampus (Fig. 5a-b and data not shown).

Statistical analysis on data from the frontal lobe area, for which we had the largest number of samples available, showed that the observed *C9orf72* reduction was significant in all the cases with the only exception of transcript 2 in *GRN* and *MAPT* mutation carriers (Additional file 1: Table S6).

We then investigated whether and to which extent the newly detected *C9orf72* transcripts were also differentially expressed in these FTD cases. In our experimental setup we could not reliably detect S4 TSS in brain samples (as it is mainly expressed in myeloid cells) and therefore we excluded it from further analysis. In agreement with our CAGEseq data we observed an increase for AS1 in the *C9orf72*-HRE patients and AS3 TSSs in the *C9orf72*-HRE and *GRN* patients as compared to controls (Fig. 5c). However only the increase for AS3 expression reached significance. Interestingly we observed a negative correlation between total *C9orf72* expression and AS3 in *C9orf72*-HRE cases (Spearman correlation -0,871).

### Aspecific neurodegenerative processes do not explain *C9orf72* reduction in FTD brains

Our finding that *C9orf72* expression is also reduced in non-HRE FTD patients suggests that the reduction of *C9orf72* expression is not entirely dependent on the repeat expansion. Sequence analysis of the GC-rich low-complexity sequence (LCS) adjacent to the HRE did not reveal any DNA variants, including the 10 bp deletion observed in up to 25 % of *C9orf72*-HRE patients [51, 52] that might explain the reduction in *C9orf72* expression; therefore we explored other potential causes. One possible explanation might be that the observed *C9orf72* decrease in *GRN* and *MAPT* cases is a consequence of the neurodegeneration process. We therefore performed a series of qPCR experiments on cDNA from brain RNA of patients with different neurodegenerative disorders including sporadic FTD, AD, ALS, PSP, PD, HD and MS patients (Table 1). In the striatum in all cases other than *C9orf72*-HRE, FTD-*MAPT* and FTD-*GRN* we did not observe a reduction in total *C9orf72* expression as compared to control brains; in contrast a trend towards an increased expression could be observed in PD cases, although the finding needs to be replicated in more samples (Additional file 1: Figure S8A). In the frontal area, sporadic FTD and AD cases showed high variability in *C9orf72* expression and no clear reduction of total *C9orf72* as compared to control brains (Additional file 1: Figure S8B). Our data therefore suggest that the *C9orf72* reduction we observed in *MAPT* and *GRN* cases is not merely due to a non-specific neurodegenerative process.

### *C9orf72*-HRE risk haplotype influences *C9orf72* expression

It has been reported that the *C9orf72*-HRE exclusively occurs within a 110 kb risk haplotype [38] common in individuals of Northern European ancestry. We investigated if the observed *C9orf72* reduction in non-HRE carriers could be explained by the risk haplotype. We first performed eQTL analysis on 119 frontal cortex control brain samples from which we have expression and genotype information. Based on the 20 SNP risk haplotype as defined by Mok et al [38], we identified seven individuals homozygous for the risk haplotype and 46 heterozygous individuals. The remaining 66 individuals were homozygous for the non-risk haplotype.

**Fig. 6:**
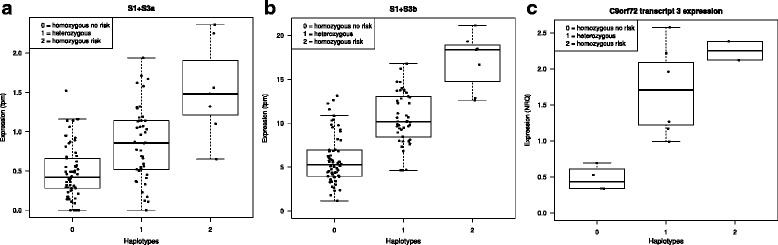
Influence of *C9orf72*-HRE risk haplotype on *C9orf72* expression. Boxplots showing the association between the *C9orf72*-HRE risk haplotype and expression in tpm for *C9orf72* S1 + S3a (**a**) and S1 + S3b (**b**) TSSs. Individuals carrying the risk haplotype in heterozygous and homozygous state have a higher expression value as compared to carriers of the non-risk haplotype. Boxplots (**c**) show quantitative comparison of *C9orf72* transcript 3 in FTD-MAPT non-carrying the risk haplotype, heterozygotes and homozygotes for the risk haplotype. Expression values are expressed as NRQs values

Our eQTL analysis shows that the risk haplotype is associated with higher expression of the *C9orf72* TSSs for transcripts 1 + 3 (FDR values 2.95eE-10 and 4.25E-20 for S1 + 3a and S1 + 3b TSSs, respectively) (Fig. 6a and b) and to lower expression of the *C9orf72* TSSs for the most abundant transcript 2 (FDR values 0.010 and 0.013 for S2A and S2B TSSs, respectively) (data not shown). On average, we observed a three fold increase in the expression of S1 + 3a and S1 + S3b TSSs in the individuals homozygous for the risk haplotype as compared to the individuals carrying the non-risk haplotype and a 1.2 and 1.5 decrease in the expression of S2a and S2b TSSs, respectively.

Next we determined by genotyping which samples used in our qPCR experiments on brain samples were carrying the risk haplotype. We identified five individuals homozygous and 34 heterozygous for the risk haplotype and 29 individuals non-carriers of the risk haplotype (Table 1).

We then correlated *C9orf72* transcripts expression levels determined by qPCR with the concordance to the risk haplotype. In our qPCR experiments we could measure the effect of the risk haplotype *C9orf72* transcript 1 and 3 in separate assays differently than with CAGEseq as *C9orf72* transcript 1 and transcript 3 share the same TSS. We observed a significant association of the risk haplotype with a higher expression of *C9orf72* transcript 3 in agreement with our CAGEseq eQTL findings (FDR value 0,0004) but not with transcript 1, with the influence of the risk haplotype on *C9orf72* transcript 3 clearly shown in Fig. 6c. We could not replicate the association of the risk haplotype with a decrease of *C9orf72* transcript 2 probably because of the small effect and sample size.

Taken together these results suggest that the risk haplotype affects *C9orf72* expression in brain tissue, but it does not fully explain the observed *C9orf72* reduction in *MAPT* and *GRN* patients as it was also detected in cases not carrying the risk allele. Therefore additional mechanisms must exist to explain the observed *C9orf72* decrease.

### *C9orf72* promoter is not methylated in *MAPT* and *GRN* mutations carriers

Several studies have shown that hypermethylation and H3 and H4 lysine trimethylation of the *C9orf72* promoter region are associated with down-regulation of *C9orf72* mRNA expression in HRE patients [26, 48, 53], with no hypermethylation observed in normal or intermediate expanded alleles (up to 43 repeats). However to the best of our knowledge, the methylation level of the *C9orf72* promoter in FTD cases with *MAPT* and *GRN* mutations has not been yet investigated.

We therefore performed an HhaI methylation-sensitive digestion assay combined with qPCR as described by Russ and colleagues [40]. Among the *C9orf72*-HRE cases, two samples were strongly hypermethylated and overall *C9orf72* promoter methylation levels were higher in *C9orf72* patients as compared to controls, *MAPT* and *GRN* mutations carriers (*p*-value 0.001644, p-Value 0.01977 and p-value 0.007349 Wilcoxon rank-sum test) while no significant differences were observed between *MAPT* cases versus controls and *GRN* cases versus controls, ruling out a direct influence of *C9orf72* methylation level on *C9orf72* expression in these patients. We then investigated whether the risk haplotype would influence the methylation level of the *C9orf72* promoter in the *MAPT* and *GRN* mutation carriers. We did not observe a significant association between the risk haplotype, in the homozygous or heterozygous state and *C9orf72* promoter methylation level (data not shown). Our results therefore suggest that *C9orf72* promoter methylation level by itself or in combination with the risk haplotype does not account for the *C9orf72* reduction observed in *MAPT* and *GRN* cases.

### Possible interaction within C*9orf72*, *MAPT* and *GRN* molecular pathways

The *C9orf72* reduction observed in *MAPT* and *GRN* mutation carriers could also imply a functional interaction within the molecular pathways disrupted by the *C9orf72*, *MAPT* and *GRN* mutations respectively. In this respect the decrease in *MAPT* transcripts containing exon 10 observed by Prudencio et al in RNAseq data from *C9orf72-HRE* patients is interesting [9]. This observation could be explained by a general impairment in RNA processing as downstream consequence of the HRE [9]. However we also observed a decrease of *C9orf72* in *MAPT* patients, which would be consistent with a functional interaction.

**Fig. 7:**
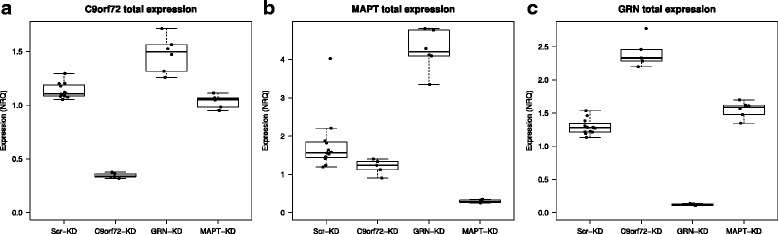
Changes in *C9orf72*, *MAPT* and *GRN* expression in BE(2)M17 cells after knockdown of *C9orf72*, *MAPT* and *GRN.* Boxplots showing changes in total *C9orf72*, *MAPT*, *GRN* relative expression values in BE(2)M17 cells after knockdown *C9orf72*, *MAPT*, *GRN* and scrambled sequences in separate experiments for N = 6. Knockdown targets are indicated on the X-axes and NRQ values are depicted on the Y-axes. **a** Decrease *C9orf72* relative expression can be observed after knockdown of *MAPT*, while silencing *GRN* results in an increase of *C9orf72* expression. **b** Decrease in *MAPT* expression is observed after knockdown of *C9orf72* and increase in *MAPT* expression after silencing *GRN*. **c** Increase in *GRN* expression is shown after knockdown of *C9orf72* and *MAPT*

To explore this possibility, we transduced BE(2) M17 human neuroblastoma cells with lentiviral constructs expressing shRNAs targeting *C9orf72* transcripts 2 and 3, *MAPT* and *GRN*. qPCR experiments on cDNAs from the transduced cells showed a clear influence on expression of *C9orf72* transcripts 2 and 3 after knock down of *MAPT* and *GRN* separately compared to scrambled controls (Fig. 7). In particular we observed a reduction of total *C9orf72* and transcript 2 and 3 when knocking down *MAPT*, while down regulation of *GRN* led to increased expression of *C9orf72*. Moreover *C9orf72* knock down revealed a decrease in *MAPT* expression and an increase of *GRN* expression, endorsing the hypothesis that *MAPT*, *GRN* and *C9orf72* interact in an a yet unknown common pathway.

### *C9orf72* reduction and abundant *C9orf72* antisense RNA foci in CD14+ monocytes of *C9orf72*-HRE patients

Our analysis of global C*9orf72* expression using CAGEseq data across the FANTOM5 collection showed that *C9orf72* is highly expressed in myeloid cells, particularly in CD14+ monocytes, in agreement with previous findings based on microarrays [54].

We performed digital PCR experiments on cDNA from CD14+ monocytes and brain RNAs from control donors and confirmed the higher expression of *C9orf72* in CD14+ monocytes (up to seven fold increase) as compared to brain (Additional file 1: Figure S7).

To investigate if the reduction of *C9orf72* observed in post mortem brain tissue of FTD cases could also be observed in monocytes, we isolated CD14+ monocytes from control donors, *C9orf72*-HRE and non familial FTD, ALS and PD patients (Table 2).

qPCR experiments on cDNAs from patients and controls for all the *C9orf72* transcripts showed a clear reduction of total *C9orf72*, transcripts 1 and 2 and AS1 in all the patients, as compared to controls (Additional file 1: Figure S9A, 9B and 9C and data not shown). In contrast transcript 3 was slightly increased in the *C9orf72*-HRE cases (Additional file 1: Figure S9D). We did not detect differences in the expression of AS2, AS3 and TSS -S4 (data not shown).

**Fig. 8:**
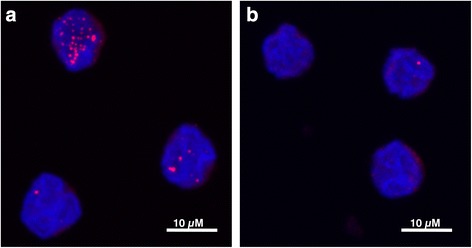
RNA foci in *C9orf72*-HRE CD14+ monocytes. **a** Antisense foci in CD14+ monocytes from the same patient detected using a TYE563-conjugated FISH probe with sequence (GGGGCC)3. **b** Sense foci in CD14+ monocytes from a *C9orf72*-HRE patient detected with a TYE563-conjugated FISH probe with sequence (CCCCGG)3. Occasionally, antisense and sense foci were observed in the same cells; however they never co-localized

Next we investigated if RNA foci were also present in CD14+ monocytes from *C9orf72*-HRE carriers. Interestingly, we could detect abundant RNA foci for the antisense *C9orf72* (Fig. 8a and Table 3) with 80-90 % of cells showing at least one nuclear focus and a small proportion of cells containing more than 20 foci. We observed RNA foci for the sense *C9orf72* as well, at least one nuclear RNA focus in up to 29 % of cells (Fig. 8b and Table 3).

**Table 3:** Summary of the RNA foci in CD14+ monocytes in *C9orf72*-HRE cases

	Patient 1 SF	Patient 2 SF	Patient 3 SF	Patient 1 ASF	Patient 2 ASF	Patient 3 ASF
Average	0.3	0.5	0.5	5.5	8.5	7.8
Median	0	0	0	3	4	4
Minimum	0	0	0	0	0	0
Maximum	10	4	6	25	38	35
%	17	29	25	90	79	85
n	72	75	106	70	132	131

The table summarizes sense and antisense foci counts from 3 patients with *C9orf72-HRE*

*SF* RNA sense foci, *ASF* RNA antisense foci

% = Percent of cells having at least one focus. No foci were detected in monocytes from an FTD patient without the repeat expansion

N = number of cells in which foci were counted automatically with a Cell Profiler speckle-counting algorithm

Overall the percentage of RNA foci for the antisense *C9orf72* in CD14+ monocytes was much higher (80–90 %) than the percentage reported for peripheral blood leukocytes (7 %) [12] and for CNS (from 9 to 26 % depending on the region) [6], while the number of RNA foci for the sense *C9orf72* is rather similar to that in the CNS [1, 5, 6, 47, 55], despite the fact *C9orf72* is much more abundant in CD14+ monocytes.

## Discussion

Our analysis on global *C9orf72* expression by using CAGEseq data reveals new and interesting features for the *C9orf72* promoter and gene locus. A major strength of CAGEseq lies in its ability to distinguish closely spaced TSS usage preference that is often tissue or cell specific. Indeed we observed several novel TSSs at the *C9orf72* locus with distinct modes of expression in a subset of myeloid cells and CNS suggesting they have a cell and/or tissue specific function. Interestingly, expression levels of S1 + S3b TSS for *C9orf72* transcripts 1 and 3 show a high correlation with the *IPO7* gene encoding for the importin 7 protein, a member of the β-karyopherin family proteins that mediate nuclear import of ribosomal proteins and export of ribosomal subunits necessary for ribosome biogenesis [44]. Currently we cannot predict whether the *C9orf72-IPO7* correlation reflects a functional interaction, but it has been shown that C9orf72 protein isoforms localize to the nuclear membrane in healthy neurons and they interact with components of the nuclear pore complex [56]. It is therefore tempting to speculate that *C9orf72* transcripts 1 and 3 in the CNS might be physiologically involved in nuclear import and export that would be affected by a decrease in their expression. This potential detrimental effect could be exacerbated in *C9orf72*-HRE patients by the presence of HRE-containing transcripts, as indicated by several studies highlighting defects in nucleocytoplasmic trafficking as results of HRE expression [10, 11, 57]. HRE expression would additionally mediate a toxic gain of function by binding and sequestering proteins involved in nucleocytoplasmic trafficking.

More research is therefore warranted in characterizing *C9orf72* functions in CNS, particularly when considering therapeutic efforts aiming at simply decreasing *C9orf72* expression to ameliorate RNA foci and DPR proteins aggregates formation.

Our CAGEseq data and qPCR results showed a consistent decrease in *C9orf72* expression in *C9orf72*-HRE patients but also in *MAPT* and *GRN* mutations carriers pointing to additional regulatory mechanisms besides the HRE-mediated *C9orf72* repression. On control brains we observed an association of the *C9orf72*-HRE risk haplotype with an increase in expression of *C9orf72* TSS for transcript 1 and 3 and decrease in TSS for transcript 2. The association of *C9orf72* expression with SNPs in the risk haplotype has been already reported in monocytes but never in brain [58, 59]. In our qPCR experiments we could detect separately *C9orf72* transcripts 1 and 3 and we replicated the association of the risk haplotype with the increase of *C9orf72* transcript 3 but not with transcript 1 or 2, likely because of the small effect and sample size. At first glance the increased expression of transcript 3 determined by the risk haplotype appears to be in contradiction with the CAGEseq and qPCR expression data showing general reduction of transcript 3 in the *C9orf72*-HRE and FTD cases with *MAPT* and *GRN* mutations compared with controls. However the increase in expression of *C9orf72* transcript 3 in individuals carrying the risk haplotype is small. Additionally, approximately half of the *GRN* and *MAPT* samples do not carry the risk haplotype.

Albeit small, the increase in expression of *C9orf72* transcript 3 might have consequences in *C9orf72*-HRE patients. The HRE can be transcribed in the pre-mRNA of abortive and/or mature *C9orf72* transcripts 1 and 3 that would accumulate in RNA foci [46, 60]. In this respect, any increase of transcripts 1 and 3 induced by the risk haplotype in *C9orf72*-HRE cases might intensify RNA foci formation although not necessarily DPR proteins aggregation as it has been suggested RAN translation might require transcripts containing the first entire intron as template [46].

The molecular reasons underlying the association of the risk haplotype with the increase in *C9orf72* transcript 3 expression are unknown. The *C9orf72*-HRE occurs on a risk haplotype covering 110 kb region between *MOB3B* and *C9orf72*. The HRE is highly polymorphic and prone to expansion particularly in the context of the risk haplotype [3]. In addition it is adjacent to a GC-rich LCS containing a 10 bp deletion that joins the repeat with the LCS region, which might promote the formation of hairpin secondary loop structures that impair DNA replication. The 10 bp deletion has been observed in up to 25 % of *C9orf72*-HRE patients [51, 52]; however it was not present in any of the *GRN* and *MAPT* mutation carriers used in this study.

Therefore, additional mechanisms must play a role in regulating *C9orf72* expression. We gathered convincing evidence for the existence of three distinct antisense transcripts head-to-head to *C9orf72* that are highly expressed in myeloid cells and to a lower extent in brain tissues in *C9orf72*-HRE cases as well as in individual without the repeat expansion. Several *C9orf7*2 antisense transcripts have been already detected in intron 1b [12, 15] and by using CAGEseq analysis we provide evidence for a longer antisense transcript that uses a more distal TSS and might extend through the HRE and accumulate in the RNA foci as shown by the slight increase in AS1 expression level by CAGEseq and qPCR in brains. The *C9orf72*-AS2 and AS3 transcripts are adjacent but downstream the HRE and they might be involved in *C9orf72* regulation.

Gene regulation by antisense transcription is rather intriguing. Antisense transcripts are transcribed from the opposite strand of protein-coding genes. This genomic arrangement immediately suggests that the sense and antisense transcripts might act on each other and increasing evidence corroborates such an hypothesis [61]. Antisense transcription has been already related to *C9orf72* regulation mainly in the context of the G-rich HRE that would mediate RNA-DNA hybrid formation (R-loops) facilitating RNA polymerase II pausing before efficient termination causing transcriptional stalling and nucleolar stress [17]. We observed a negative correlation between *C9orf72* sense transcripts and antisense transcript AS3 in brain tissues from *C9orf72-HRE* patients and therefore it might be relevant to investigate whether the novel antisense transcripts hamper or amplify the HRE antisense effects. In addition increasing evidence shows that antisense transcripts act at nearly every level of gene regulation (transcription, mRNA processing and translation) and even function simultaneously at multiple steps. It has been shown that antisense expression can regulate transcription by affecting DNA methylation. Several studies, including our own, show that hypermethylation of the CpG-island at the 5′ end of the repeat contributes to *C9orf72* repression in a high of *C9orf72*-HRE carriers, yet down-regulation of *C9orf72* is more prevalent and shown among FTD sporadic patients [19] and *MAPT* and *GRN* mutation carriers (this study). Very recently, co-localization of para-nucleolar DPR proteins inclusions with heterochomatin and H3K9me2 [18], a marker of transcriptional repression linked to R-loop-induced transcriptional silencing, has been reported [62]. In light of these findings it would be interesting to determine whether and to what extent the *C9orf72* antisense AS3 affects histone modifications at the *C9orf72* locus not only in *C9orf72*-HRE but also in *MAPT* and *GRN* patients.

The robust *C9orf72* expression in myeloid cells and the changes in *C9orf72* expression in CD14+ monocytes after exposure to microbial pathogens suggest that *C9orf72* might be involved in immune-related processes, in agreement also with the recent findings that in mice is required for normal function of myeloid cells [63]. Moreover it has been proposed that *C9orf72* might play a role in the initial steps of autophagy as suggested by its interaction with FIP200, a component of the ULK1-ATG13 autophagosome initiation complex [64].

Consistent with a possible *C9orf72* role in autophagy, neurons differentiated from patients-derived iPSC, are more sensitive to autophagy inhibitors and presented higher p62 levels suggesting autophagy might be compromised [47].

The half-life of monocytes in blood is relatively short: approximately 1 day in mice and 3 days in human. Once monocytes are stimulated by an inflammatory response they activate pro-survival pathways, migrate to tissues where they mediate direct antimicrobial activity by releasing tumor necrosis factor, chemokines, engage in phagocytosis and differentiate to macrophages through molecular mechanisms that involve autophagy [65].

While waiting for further research to clarify the exact role of *C9orf72* in the autophagy process it is tempting to speculate that reduced *C9orf72* expression might impair autophagy affecting monocyte-macrophage differentiation and thereby hamper host inflammatory responses. Although our findings will need to be replicated in a larger samples size including age-matched controls, our qPCR results on CD14+ monocytes from *C9orf7*-HRE carriers and from patients with clinical diagnosis of FTD and ALS are in line with this hypothesis. Currently we do not have evidence that monocytes in these patients are impaired but we show that they carry a very high antisense RNA foci burden.

## Conclusion

In conclusion, our findings strongly imply that several mechanisms acting independently from the HRE, or in a concerted manner, contribute to regulate *C9orf72* expression. We showed that the decrease in *C9orf72* expression is a widespread phenomenon in FTD pathogenesis suggesting *C9orf72* plays a more general role in neurodegeneration. Moreover the *C9orf72* TSSs profile in CNS and myeloid cells suggests distinct *C9orf72* transcripts have tissue specific function and they call attention to a potential *C9orf72* role in immune response.

## Additional file

Additional file 1: Supplementary Data. **Figure S1:** Definition of TSSs at the *C9orf72* locus; **Figure S2:** *C9orf72* expression changes in challenged CD14+ monocytes. **Figure S3:** Distinct TSSs expression at *C9orf72* locus in CNS, myeloid and lymphoid cells. **Figure S4:** Sense and antisense *C9orf72* transcripts at the *C9orf72* locus. **Figure S5:** Distinct *C9orf72* TSSs expression in control brains. **Figure S6:** *C9orf72* expression in adult and fetal cortex. **Figure S7:** *C9orf72* expression level in CD14+ monocytes, brain tissue and microglia. **Figure S8:** *C9orf72* expression in brains of patients with different neurodegenerative diseases. **Figure S9:** *C9orf72* expression in CD14+ monocytes. **Suppl. excel File 1:** WGCNA results. **Suppl. excel File 2:** Correlations values between *C9orf72* TSS(s) and all the other TSSs in the co-expressed modules. **Table S1:** Expression of *C9orf72* TSSs as defined in CAGEseq dataset 1. **Table S2:** Expression of *C9orf72* TSSs as defined in CAGEseq dataset 2. **Table S3:** List of primers used in this study. **Table S4:** Biological functions significant to the three modules related to *C9orf72* TSSs as identified by WGCNA. **Table S5:** Biological functions significant for genes that correlate with *C9orf72* TSSs in the WGCNA identified modules. **Table S6:** Summary of the Mann-Whitney test performed on NRQ values from qPCR experiments on medial frontal gyrus. (ZIP 20675 kb)
