# Supplementary material for: C9orf72 is differentially expressed in the central nervous system and myeloid cells and consistently reduced in C9orf72, MAPT and GRN mutation carriers
Source: Acta Neuropathol Commun. 2016 Apr 14;4:37. doi: 10.1186/s40478-016-0306-7 (PMC4832459; doi:10.1186/s40478-016-0306-7)
Supplement: Additional file 1: — Supplementary Data. Figure S1: Definition of TSSs at the C9orf72 locus; Figure S2: C9orf72 expression changes in challenged CD14+ monocytes. Figure S3: Distinct TSSs expression at C9orf72 locus in CNS, myeloid and lymphoid cells. Figure S4: Sense and antisense C9orf72 transcripts at the C9orf72 locus. Figure S5: Distinct C9orf72 TSSs expression in control brains. Figure S6: C9orf72 expression in adult and fetal cortex. Figure S7: C9orf72 expression level in CD14+ monocytes, brain tissue and microglia. Figure S8: C9orf72 expression in brains of patients with different neurodegenerative diseases. Figure S9: C9orf72 expression in CD14+ monocytes. Suppl. excel File 1: WGCNA results. Suppl. excel File 2: Correlations values between C9orf72 TSS(s) and all the other TSSs in the co-expressed modules. Table S1: Expression of C9orf72 TSSs as defined in CAGEseq dataset 1. Table S2: Expression of C9orf72 TSSs as defined in CAGEseq dataset 2. Table S3: List of primers used in this study. Table S4: Biological functions significant to the three modules related to C9orf72 TSSs as identified by WGCNA. Table S5: Biological functions significant for genes that correlate with C9orf72 TSSs in the WGCNA identified modules. Table S6: Summary of the Mann-Whitney test performed on NRQ values from qPCR experiments on medial frontal gyrus. (ZIP 20675 kb) [file 40478_2016_306_MOESM1_ESM.zip › Supplementary data 22March2016.pdf]

C9orf72 is differentially expressed in the central nervous system and myeloid cells and consistently reduced in *C9orf72*, *MAPT* and *GRN* mutation carriers

Patrizia Rizzu,[1,3,*] Cornelis Blauwendraat,[1,3] Sasja Heetveld,[1,3] Emily M. Lynes,[1] Melissa Castillo-Lizardo,[1] Ashutosh Dhingra,[1] Elwira Pyz,[1] Markus Hobert[1,2]Matthis Synofzik[1,2], Javier Simón-Sánchez, [1,2] Margherita Francescatto,[1] Peter Heutink[1,2]

[3] These authors contributed equally to this work

*To whom correspondence should be addressed:

Patrizia Rizzu

German Center for Neurodegenerative Diseases (DZNE)

Otfried-Müller Strasse 23, Tübingen, 72076 Germany

Email: patrizia.rizzu@dzne.de

Tel: 004970719254082

Inventory of Supplementary Data

Supplementary Data include 9 figures, 6 tables and 2 supplementary files.

Suppl. Fig1 Definition of TSSs at the *C9orf72* locus

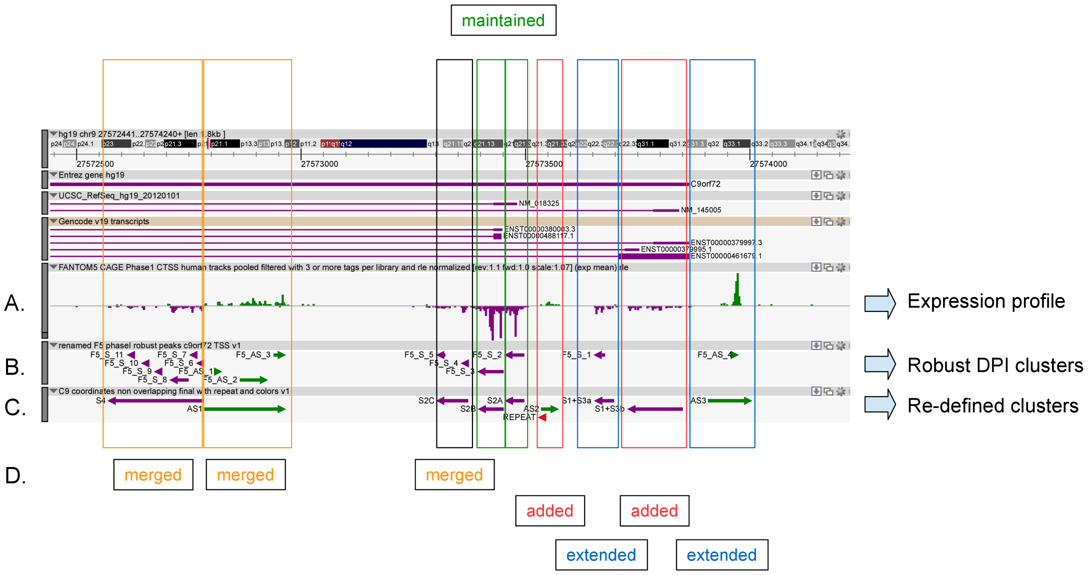

merged: several FANTOM5 clusters merged into one
extended: the original FANTOM5 cluster was extended to include all CAGEseq signal in a stretch
added: a new cluster was added in a place where CAGEseq signal was visible but no DPI cluster was available
maintained: the original FANTOM5 cluster is maintained without modifications

Graphical representation of the connection between DPI clusters expression profiles and TSSs used to describe transcriptional events at *C9orf72* 5' end. (A) FANTOM5 human CAGEseq expression profile at *C9orf72* 5' end. (B) Human robust DPI clusters at the *C9orf72* 5' end as defined in the context of FANTOM5 promoterome project. (C) *C9orf72* TSSs redefined according to the procedure described in the methods. (D) Modifications made on the robust DPI clusters to obtain the final list of *C9orf72* TSSs used in this work.

Suppl. Fig.2 *C9orf72* expression changes in challenged CD14+ monocytes

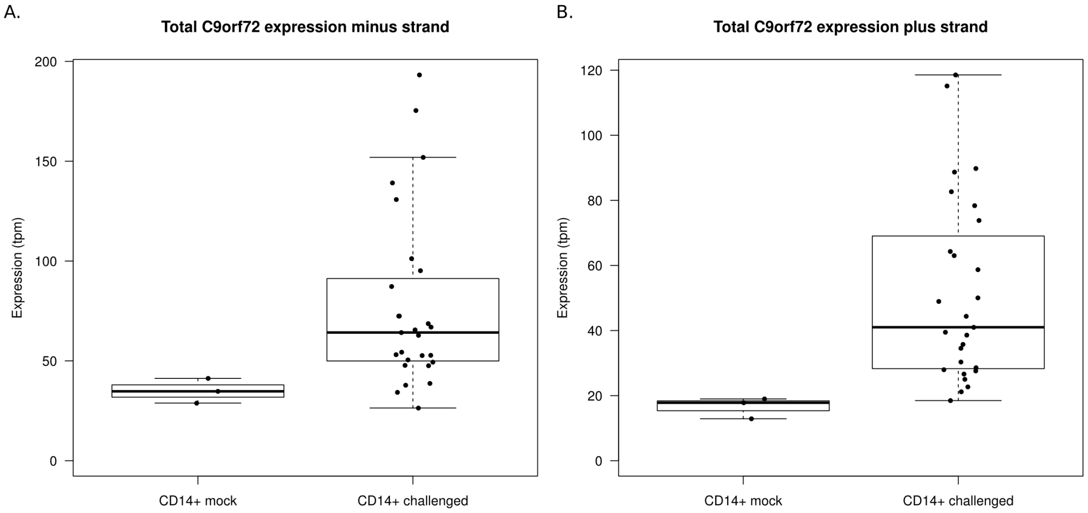

Total *C9orf72* expression differences (A) in the sense and (B) antisense strand in CD14+ monocytes challenged with B-glucan, interpheron plus N-hexane, lipopolysaccharide, threalose dimycolate, *Candida*, *Cryptococcus*, *Streptococcus* group A and *Salmonella* and in CD14+ monocytes mock-challenged. Overall *C9orf72* expression is increased in CD14+ monocytes after challenging as compared to CD14+ mock-treated. *C9orf72* expression values expressed in tpm were obtained from FANTOM5 CAGEseq data.

Suppl. Fig.3 Distinct TSSs expression at *C9orf72* locus in CNS, myeloid and lymphoid cells

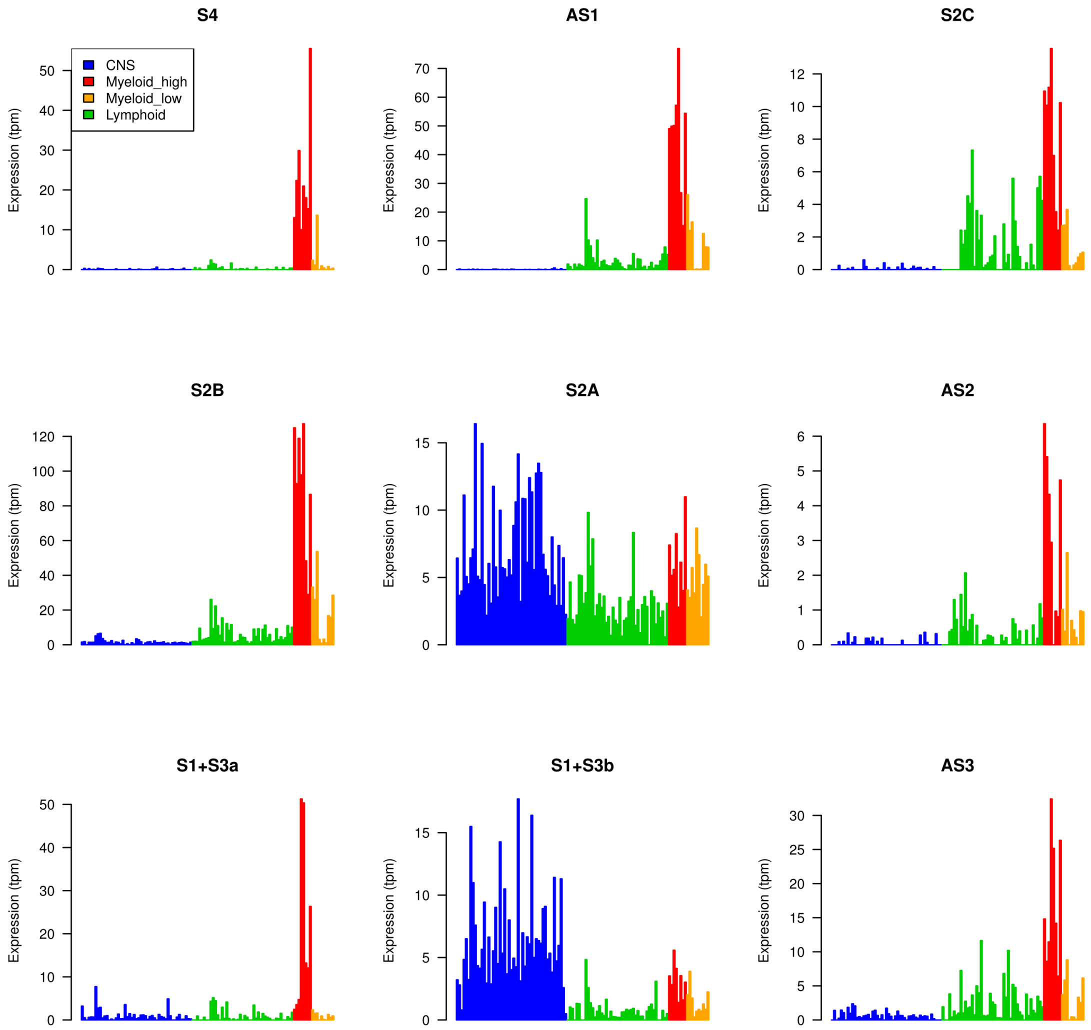

Expression values for the distinct TSSs at the *C9orf72* locus are indicated in tpm. CD14+ monocytes, eosinophils and neutrophils show in general the highest expression and they are here indicated as myeloid-high. *C9orf72* TSSs expression is lower in other myeloid cells like mast cells and macrophages (myeloid-low) and lymphoid cells. TSSs expression at the *C9orf72* locus in CNS is also moderate and overall comparable with myeloid-low and lymphoid cells with the exception of S2a TSS highly expressed in CNS and at comparable level with myeloid-high

and the S1+S3b TSS that is mainly expressed in CNS. *C9orf72-AS* TSSs show higher expression in myeloid and lymphoid cells.

Suppl. Fig.4 Sense and antisense ***C9orf72*** **transcripts at the** ***C9orf72*** **locus**.

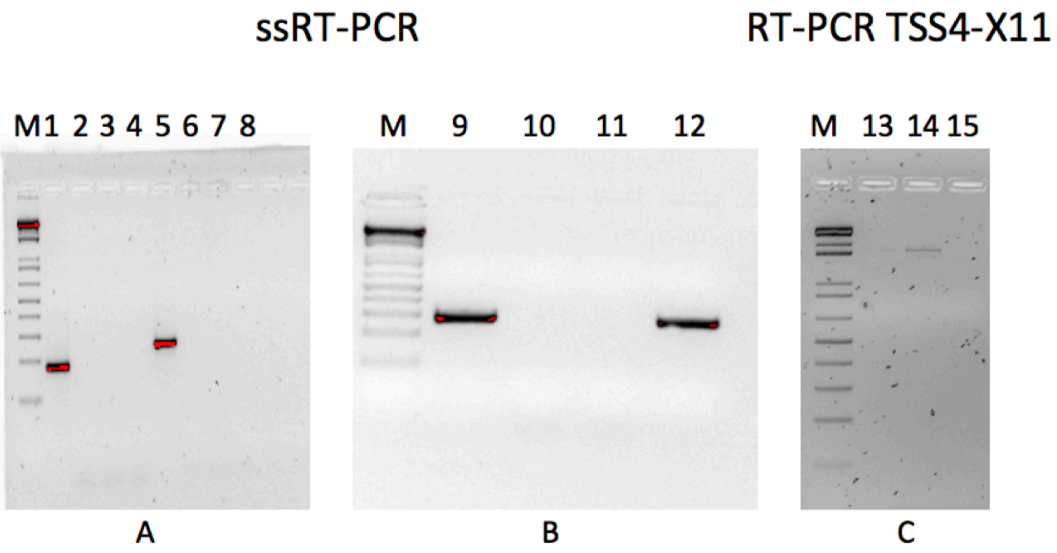

The specificity of the novel antisense transcripts was confirmed by ssRT-PCR experiments showing a product on agarose gel only in the specific ssRT reactions after two PCR rounds: panel A lane 1 for AS1 and lane 5 for AS3 and panel B lanes 9 and 12 for AS2. No amplification was observed in the additional RT reactions set up with (panel A lanes 2 and 6 and panel B lane 10) and without oligodT and random decamers (panel A lanes 4 and 7 and panel B lane 11) or in the water template (lanes 4 and 8). The following primers combinations were used for the ssRT-PCR experiments: for AS1 single strand specific cDNA was synthesized using LK-ssRT-AS1 followed by two PCR rounds with LK and ssRT-AS1R and AS1F-short with ssRT-AS1R; for AS2 single strand specific cDNA was synthesized using LK-ssRT-AS2 followed by two PCR rounds with LK and ssRT-AS2R and AS2F-short with ssRT-AS2R; for AS3 single strand specific cDNA was synthesized using LK-ssRT-AS3 followed by two PCR rounds with LK and ssRT-AS3R and AS3F-short with ssRT-AS3R.

Panel C shows the amplified *C9orf72* novel transcript 5 containing the putative exon 1c using the primer combination TSS4-X11utr-R in brain (lane 13) and in CD14+ monocytes (lane 14). Lane 15: water template negative control. M: 1kb+ DNA ladder.

Suppl. Fig.5 Distinct *C9orf72* TSSs expression in control brains

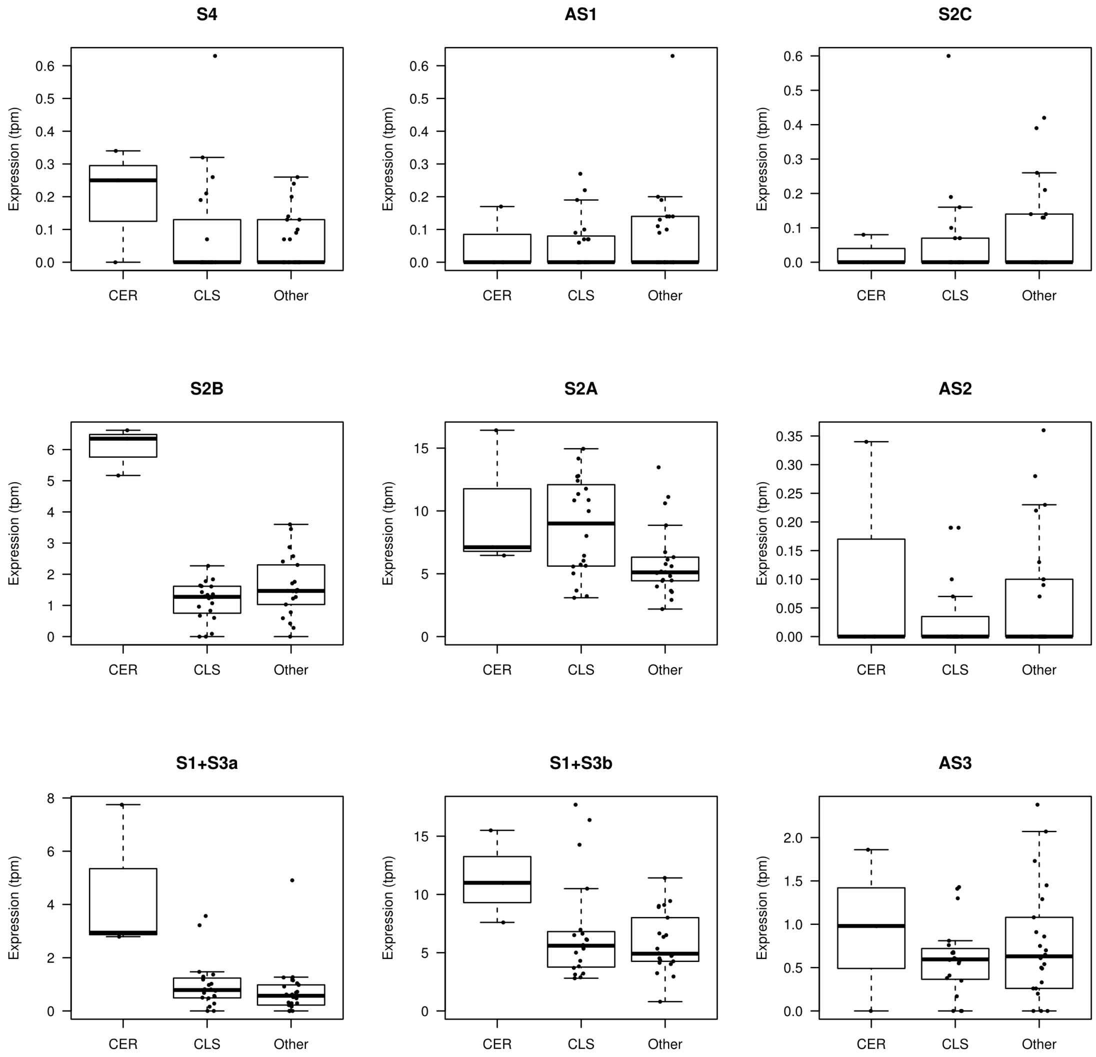

(A) Boxplots showing regional TSSs expression levels indicated in tpm at the *C9orf72* locus in adult CNS from FANTOM5 CAGEseq data: cerebellum (CER), cerebral cortex and limbic system (CLS), striatum, basal ganglia, thalamus (others). In general we can observe *C9orf72* is highly expressed in cerebellum. S2A and S1+S3b TSSs show to be highly expressed in all the CNS while the ASs TSSs are barely detectable.

Suppl. Fig.6 *C9orf72* expression in adult and fetal cortex

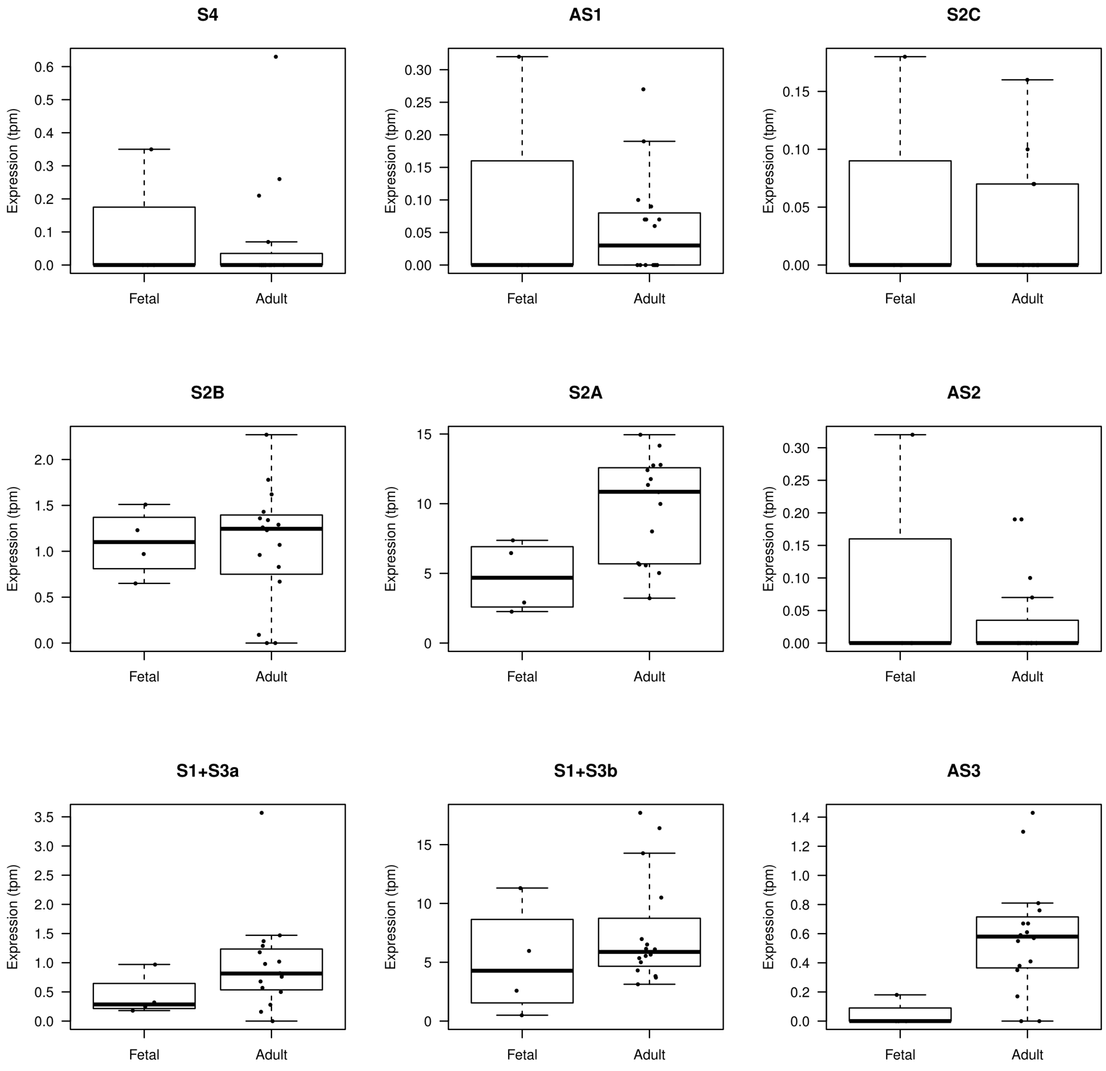

Boxplots showing TSSs expression levels in fetal and adult cortex at the *C9orf72* locus. No major differences can be observed among *C9orf72* expression levels in fetal and adult cortex with the exception of a slight increase in S2a and AS3 TSSs expression values. Expression values are defined in tpm.

Suppl. Fig. 7 *C9orf72* expression level in CD14+ monocytes, brain tissue and microglia.

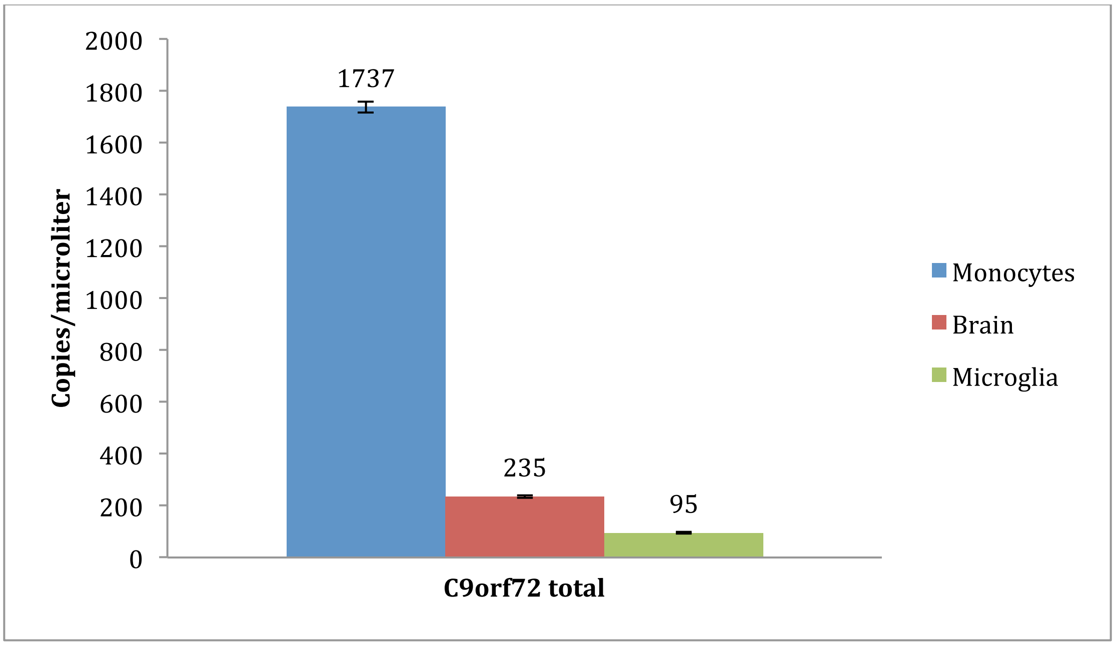

Bar chart showing *C9orf72* expression level in human CD14+ monocytes, brain (medial frontal gyrus) and microglia cells from donors with no neurodegenerative disorders expressed in copies/microliter as determined in digital PCR experiments using a Taqman assay targeting *C9orf72* transcript 1, 2 and 3. Each measurement was performed in duplicate and the error bars reflect the standard deviations between replicates.

Suppl. Fig.8 *C9orf72* expression in brains of patients with different neurodegenerative diseases

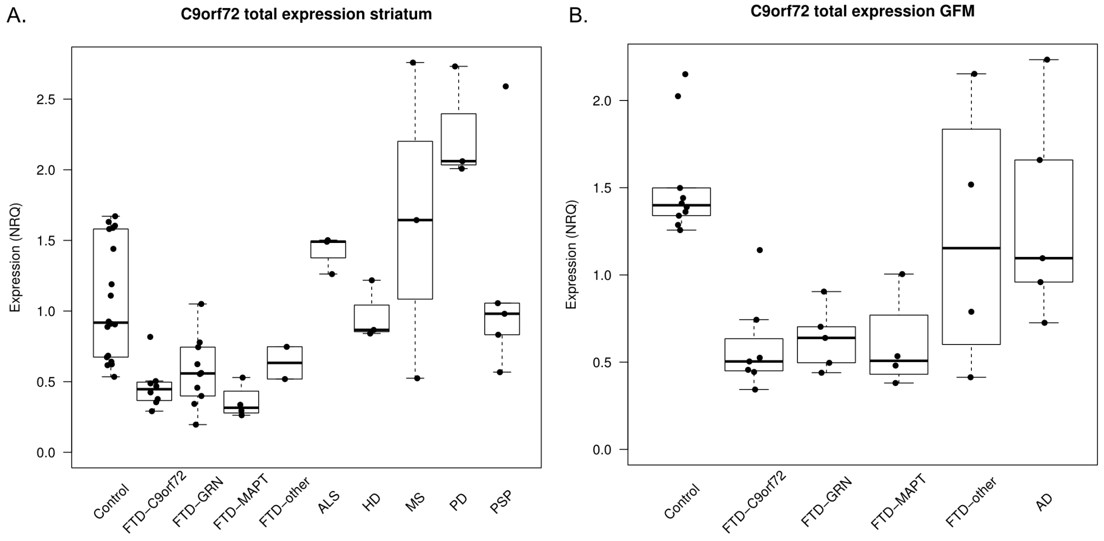

(A) Boxplots showing relative expression values in *C9orf72* total in brains from control donors, *C9orf72*-HRE, FTD-*GRN* and FTD-*MAPT* mutation carriers, FTD sporadic, ALS, HD, MS, PD and PSP patients in the caudate/putamen area. Small *C9orf72* decrease can be observed in *C9orf72*-HRE, *MAPT* and *GRN* mutation carriers as compared to controls, while a trend of *C9orf72* increased expression is detected in PD samples. (B) Boxplots showing relative expression values in *C9orf72* total in brains of *C9orf72*-HRE, *MAPT* and *GRN* mutation carriers, FTD sporadic and AD patients in medial frontal gyrus. AD and FTD sporadic patients show high variability in *C9orf72* expression.

Suppl. Fig.9 *C9orf72* expression in CD14+ monocytes

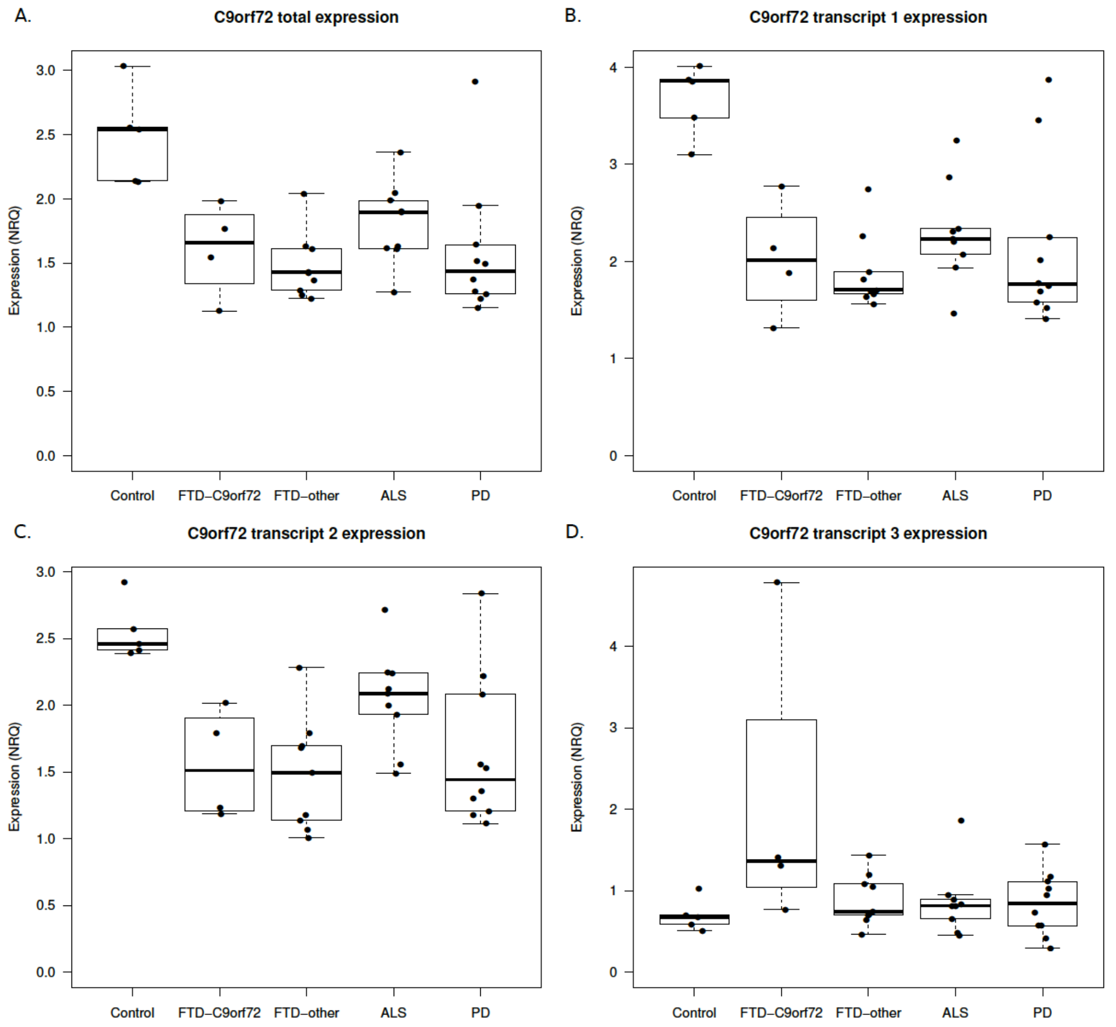

Quantitative comparisons of *C9orf72* expression in CD14+ monocytes isolated from control donors, ALS, *C9orf72*-HRE, FTD and PD cases for *C9orf72* total (**A**), transcript 1(**B**) and transcript 2 (**C**) and transcript 3 (**D**). Although high variation in *C9orf72* expression, reduction can be observed in all cases as compared to control donors, for *C9orf72* total and transcripts 1 and 2 but not transcript 3. Expression values are expressed as NRQs values and measurements are performed in triplicates.

Suppl. File 1 WGCNA results

Columns in file 1 indicate a) clusterID (tag cluster ID assigned to each tag cluster in the clustering process; b)geneSymbol (to which annotated gene the cluster maps to); c)moduleColor (module to which the gene was assigned); d-am) pairs of columns containing module membership (MM) and corresponding p-value for each TSS included in the analysis and for each module.

Suppl. File 2 Correlations values between *C9orf72* TSS(s) and all the other TSSs in the co-expressed modules.

The excel file provides Spearman correlation values between each of the C9orf72 TSSs and all the other TSSs of protein coding genes assigned to the same module (turquoise, yellow, blue and pink modules). Correlations were calculated as described in detail in material and methods.

Suppl. Table 1 Expression of *C9orf72* TSSs as defined in CAGEseq dataset 1

group	experiment_name	S4.minus	AS1.plus	S2C.minus	S2B.minus	S2A.minus	AS2.plus	S1.S3a.minus	S1.S3b.minus	AS3.plus
CNS	amygdala - adult, donor10196 : CNhs13793 ctss	0	0	0	1,61	6,44	0	3,22	3,22	0
CNS	amygdala, adult, donor10252 : CNhs12311 ctss	0,32	0,22	0	1,84	3,67	0	0,54	2,81	1,41
CNS	brain, adult, donor1 : CNhs11796 ctss	0	0	0	0	3,99	0	0	0,8	0
CNS	brain, adult, pool1 : CNhs10617 ctss	0,26	0	0,26	1,5	11,12	0,09	0,62	4,85	0,26
CNS	caudate nucleus - adult, donor10196 : CNhs13802 ctss	0	0	0	1,45	5,07	0	0,72	6,51	1,45
CNS	caudate nucleus, adult, donor10252 : CNhs12321 ctss	0,1	0,1	0	1,47	4,52	0,1	0,69	3,24	1,08
myeloidChallenged	CD14+ monocytes - mock treated, donor1 : CNhs13468 ctss	6,14	9,01	1,23	22,39	4,51	2,05	3	3,96	7,92
myeloidChallenged	CD14+ monocytes - mock treated, donor2 : CNhs13484 ctss	5,02	5,73	1,07	15,05	5,37	1,07	2,51	5,73	6,09
myeloidChallenged	CD14+ monocytes - mock treated, donor3 : CNhs13491 ctss	3,75	2,81	1,41	12,2	6,57	1,41	1,41	3,52	13,6
myeloidChallenged	CD14+ monocytes - treated with B-glucan, donor1 : CNhs13474 ctss	19,94	20,11	3,49	34,9	5,32	2,33	3,49	5,32	13,3
myeloidChallenged	CD14+ monocytes - treated with B-glucan, donor2 : CNhs13489 ctss	18,6	17,74	2,86	22,89	5,72	1,43	6,58	8,87	20,32
myeloidChallenged	CD14+ monocytes - treated with B-glucan, donor3 : CNhs13495 ctss	16,58	17,19	5,73	25,58	4,91	2,46	5,73	8,39	43,38
myeloidChallenged	CD14+ monocytes - treated with BCG, donor1 : CNhs13465 ctss	30,16	18,52	11,17	35,21	3,52	2,91	2,76	4,44	22,96
myeloidChallenged	CD14+ monocytes - treated with BCG, donor2 : CNhs13475 ctss	20,81	18,84	10,94	27,49	4,71	1,06	2,58	5,92	21,11
myeloidChallenged	CD14+ monocytes - treated with BCG, donor3 : CNhs13543 ctss	9,69	19,13	10,19	24,41	4,78	4,66	2,01	3,27	40,52
myeloidChallenged	CD14+ monocytes - treated with Candida, donor1 : CNhs13473 ctss	73,13	32,76	22,7	72,84	6,18	5,17	7,76	10,63	51,87
myeloidChallenged	CD14+ monocytes - treated with Candida, donor2 : CNhs13488 ctss	47,24	26,42	7,15	45,69	5,59	1,86	9,63	15,54	54,39
myeloidChallenged	CD14+ monocytes - treated with Candida, donor3 : CNhs13494 ctss	46,18	13,37	8,51	57,12	4,25	2,43	11,55	11,55	62,59
myeloidChallenged	CD14+ monocytes - treated with Cryptococcus, donor1 : CNhs13472 ctss	15,11	16,07	4,4	30,09	7,14	4,26	5,77	6,18	18,27
myeloidChallenged	CD14+ monocytes - treated with Cryptococcus, donor2 : CNhs13487 ctss	8,41	6,9	2,37	18,98	6,47	1,08	6,04	7,12	19,62
myeloidChallenged	CD14+ monocytes - treated with Cryptococcus, donor3 : CNhs13546 ctss	5,72	7,1	3,29	22,52	7,1	1,91	6,58	2,42	49,71
myeloidChallenged	CD14+ monocytes - treated with Group A streptococci, donor1 : CNhs13469 ctss	65,5	31,1	22,85	70,92	3,53	4,24	7,19	5,42	53,37
myeloidChallenged	CD14+ monocytes - treated with Group A streptococci, donor2 : CNhs13532 ctss	20,89	22,19	17,99	43,22	4,06	2,76	6,67	8,41	90,22
myeloidChallenged	CD14+ monocytes - treated with Group A streptococci, donor3 : CNhs13492 ctss	46,45	23,23	29,03	59,52	3,39	3,63	9,44	4,11	91,7
myeloidChallenged	CD14+ monocytes - treated with IFN + N-hexane, donor1 : CNhs13466 ctss	6,01	9,15	1,83	14,64	5,49	4,97	2,09	4,18	7,06
myeloidChallenged	CD14+ monocytes - treated with IFN + N-hexane, donor2 : CNhs13476 ctss	12,36	13,26	4,17	20,41	4,17	2,23	2,53	6,85	9,53
myeloidChallenged	CD14+ monocytes - treated with IFN + N-hexane, donor3 : CNhs13490 ctss	7,68	10,69	2,67	17,03	4,01	2,67	2	5,34	9,35
myeloidChallenged	CD14+ monocytes - treated with lipopolysaccharide, donor1 : CNhs13470 ctss	7,16	10,18	1,7	13,01	1,32	1,51	1,51	1,7	6,79
myeloidChallenged	CD14+ monocytes - treated with lipopolysaccharide, donor2 : CNhs13533 ctss	5,79	13,07	4,8	24,64	3,64	0,66	4,13	4,8	20,84
myeloidChallenged	CD14+ monocytes - treated with lipopolysaccharide, donor3 : CNhs13545 ctss	6,07	7,47	4,05	19,36	3,04	1,77	1,39	3,92	18,73
myeloidChallenged	CD14+ monocytes - treated with Salmonella, donor1 : CNhs13471 ctss	10,58	16,9	3,45	30,93	2,64	3,22	3,22	2,3	10,23
myeloidChallenged	CD14+ monocytes - treated with Salmonella, donor2 : CNhs13485 ctss	10,66	13,52	3,38	27,31	3,9	1,04	3,64	3,9	14,04
myeloidChallenged	CD14+ monocytes - treated with Salmonella, donor3 : CNhs13493 ctss	9,13	10,96	3,39	27,13	4,17	1,83	3,65	5,22	13,83
myeloidChallenged	CD14+ monocytes - treated with Trehalose dimycolate (TDM), donor1 : CNhs13467 ctss	27,33	20,71	8,49	39,69	4,03	5,47	6,62	9,06	23,87
myeloidChallenged	CD14+ monocytes - treated with Trehalose dimycolate (TDM), donor2 : CNhs13483 ctss	17,54	19,23	6,22	22,91	3,39	3,11	7,07	7,07	26,59
myeloidChallenged	CD14+ monocytes - treated with Trehalose dimycolate (TDM), donor3 : CNhs13544 ctss	11,27	20,33	8,42	29,14	4,66	1,81	4,79	4,53	51,67
myeloid_high	CD14+ Monocytes, donor1 : CNhs10852 ctss	13,06	49,08	10,95	124,99	7,41	6,36	2,47	3,53	14,83
myeloid_high	CD14+ Monocytes, donor2 : CNhs11954 ctss	22,38	49,92	10,08	92,84	5,16	5,41	3,57	2,83	8,61
myeloid_high	CD14+ Monocytes, donor3 : CNhs11997 ctss	29,9	50,15	11,18	118,88	5,59	4,33	4,75	5,59	11,46
CNS	cerebellum - adult, donor10196 : CNhs13799 ctss	0	0	0	5,17	6,46	0	7,75	15,5	0
CNS	cerebellum, adult, donor10252 : CNhs12323 ctss	0,34	0,17	0,08	6,35	7,11	0,34	2,79	11	1,86
CNS	cerebellum, adult, pool1 : CNhs11795 ctss	0,25	0	0	6,62	16,42	0	2,94	7,6	0,98
CNS	corpus callosum, adult, pool1 : CNhs10649 ctss	0,2	0,14	0,14	3,6	5,09	0,07	0,48	4,35	2,38
CNS	diencephalon, adult : CNhs12610 ctss	0	0	0	2,3	4,83	0,23	0,92	4,14	2,07
myeloid_high	Eosinophils, donor2 : CNhs12548 ctss	10,02	57,19	13,56	97,87	8,25	2,95	51,29	4,13	32,43
myeloid_high	Eosinophils, donor3 : CNhs12549 ctss	20,99	76,95	7	127,32	2,8	0	50,37	1,4	25,18
CNS	frontal lobe, adult, pool1 : CNhs10647 ctss	0	0,06	0	1,34	14,95	0	1,02	5,66	0,38
CNS	globus pallidus - adult, donor10196 : CNhs13801 ctss	0	0	0	1,49	4,47	0	0	9,44	0,5
CNS	globus pallidus, adult, donor10252 : CNhs12319 ctss	0	0	0	2,41	2,19	0	0,22	2,96	0,33
CNS	hippocampus - adult, donor10196 : CNhs13795 ctss	0	0	0,6	0,6	6,04	0	0	6,64	0,6
CNS	hippocampus, adult, donor10252 : CNhs12312 ctss	0,19	0	0,19	1,64	3,09	0,19	0,48	2,89	0,68
CNS	insula, adult, pool1 : CNhs10646 ctss	0	0,19	0	1,43	11,77	0,19	1,37	5,54	0,81
CNS	locus coeruleus - adult, donor10196 : CNhs13808 ctss	0	0,2	0	0,59	5,78	0,1	0,29	9,02	0,2
CNS	locus coeruleus, adult, donor10252 : CNhs12322 ctss	0	0,11	0	2,58	3,55	0,22	0,32	4,52	1,29
CNS	medial frontal gyrus - adult, donor10196 : CNhs13796 ctss	0	0	0	0	9,99	0	3,57	14,27	1,43
CNS	medial frontal gyrus, adult, donor10252 : CNhs12310 ctss	0	0,1	0,1	0,67	5,73	0,1	0,76	5,35	0,67
CNS	medial temporal gyrus - adult, donor10196 : CNhs13809 ctss	0,26	0	0	0,09	5,64	0	1,47	10,5	0,17
CNS	medial temporal gyrus, adult, donor10252 : CNhs12316 ctss	0	0,09	0	1,23	5,03	0,19	0,57	3,7	0,76
CNS	medulla oblongata - adult, donor10196 : CNhs13800 ctss	0	0	0,42	0,42	6,32	0	1,26	8,01	0
CNS	medulla oblongata, adult, donor10252 : CNhs12315 ctss	0	0,19	0	3,45	5,18	0	0,29	4,03	1,73
CNS	medulla oblongata, adult, pool1 : CNhs10645 ctss	0,13	0,13	0,13	2,87	8,86	0	0,52	4,95	0,91
myeloid_high	Neutrophils, donor1 : CNhs10862 ctss	18,07	26,78	3,55	48,39	6,13	0,97	13,23	3,55	14,19
myeloid_high	Neutrophils, donor2 : CNhs11959 ctss	15,3	15,3	2,42	29	4,03	0,81	12,08	1,61	6,44
myeloid_high	Neutrophils, donor3 : CNhs11905 ctss	55,53	54,4	10,24	86,62	10,99	4,74	26,35	3,03	26,35
CNS	nucleus accumbens, adult, pool1 : CNhs10644 ctss	0,07	0	0	1,76	10,61	0	1,15	4,26	0,54
CNS	occipital cortex - adult, donor10196 : CNhs13798 ctss	0	0	0	0	14,17	0	1,18	17,71	0
CNS	occipital cortex, adult, donor10252 : CNhs12320 ctss	0	0	0	1,07	3,22	0	0,98	3,13	0,59
CNS	occipital lobe, adult, donor1 : CNhs11787 ctss	0	0	0,16	1,62	10,87	0	0,16	6,98	1,3
CNS	occipital pole, adult, pool1 : CNhs10643 ctss	0	0,07	0	1,78	10,84	0	0,82	4,31	0,67
CNS	olfactory region, adult : CNhs12611 ctss	0,13	0	0,39	0,78	6,13	0,13	1,04	6,66	0,65

CNS	paracentral gyrus, adult, pool1 : CNhs10642 ctss	0,21	0,07	0,07	2,27	12,41	0	0,5	6,1	0,35
CNS	parietal lobe - adult, donor10196 : CNhs13797 ctss	0,63	0	0	1,26	11,35	0	0	16,4	0
CNS	parietal lobe, adult, donor10252 : CNhs12317 ctss	0	0	0	1,29	5,58	0	1,29	5,01	0,57
CNS	parietal lobe, adult, pool1 : CNhs10641 ctss	0,07	0	0,07	1,36	12,75	0	0,81	6,51	0,61
CNS	pons, adult, pool1 : CNhs10640 ctss	0,07	0,14	0,21	1,71	13,48	0	0,62	6,36	0,75
CNS	postcentral gyrus, adult, pool1 : CNhs10638 ctss	0	0,07	0,07	0,83	12,79	0	0,28	6,15	0,41
CNS	putamen, adult, donor10196 : CNhs12324 ctss	0	0	0,13	1,03	6,72	0	4,91	8,92	0,26
CNS	spinal cord - adult, donor10196 : CNhs13807 ctss	0,14	0,14	0,14	0,28	5,6	0,28	0,98	9,1	0,7
CNS	spinal cord, adult, donor10252 : CNhs12227 ctss	0,24	0	0	1,22	5,13	0	0	4,88	0,49
CNS	substantia nigra, adult, donor10252 : CNhs12318 ctss	0	0	0	1,46	3,65	0,36	0	5,35	0,61
CNS	temporal lobe, adult, pool1 : CNhs10637 ctss	0	0,27	0,07	0,96	8,01	0,07	0,68	3,83	0,55
CNS	thalamus - adult, donor10196 : CNhs13794 ctss	0	0,63	0	1,27	4,44	0	1,27	11,42	0
CNS	thalamus, adult, donor10252 : CNhs12314 ctss	0,09	0,09	0	1,46	2,92	0	0,17	4,73	0,86
lymphoid	CD19+ B Cells (pluriselect), donor090309, donation1 : CNhs12177 ctss	0	1,94	0	1,94	1,94	0	0	0	1,94
lymphoid	CD19+ B Cells (pluriselect), donor090309, donation2 : CNhs12179 ctss	0,52	1,04	0	2,07	4,66	0	0	1,04	0
lymphoid	CD19+ B Cells (pluriselect), donor090309, donation3 : CNhs12181 ctss	0	0	0	1,87	1,87	0	0,93	0,93	0
lymphoid	CD19+ B Cells (pluriselect), donor090325, donation1 : CNhs12531 ctss	0,38	1,91	0	9,55	1,53	0,38	0	0	3,82
lymphoid	CD19+ B Cells (pluriselect), donor090325, donation2 : CNhs12175 ctss	0	0,88	0	2,65	2,21	0,44	0	1,33	0,44
lymphoid	CD19+ B Cells (pluriselect), donor090612, donation1 : CNhs12183 ctss	0	1,94	0	3,24	5,18	1,3	0,65	1,3	1,3
lymphoid	CD19+ B Cells (pluriselect), donor090612, donation2 : CNhs12185 ctss	0	1,46	0	3,66	5,13	0,73	0	0	0,73
lymphoid	CD19+ B Cells (pluriselect), donor090612, donation3 : CNhs12188 ctss	1,02	1,02	0	4,08	3,06	0	0	0	1,02
lymphoid	CD19+ B Cells, donor1 : CNhs12343 ctss	2,42	24,68	2,42	26,14	3,87	1,45	4,36	4,84	7,26
lymphoid	CD19+ B Cells, donor2 : CNhs12352 ctss	1,55	10,34	1,55	9,31	9,83	0,52	5,17	2,59	1,03
lymphoid	CD19+ B Cells, donor3 : CNhs12354 ctss	1,38	8,26	2,41	22,37	5,85	2,07	4,47	1,38	2,75
lymphoid	CD4+ T Cells, donor1 : CNhs10853 ctss	0,2	4,13	4,52	11,01	7,87	0,39	1,18	0,98	1,77
lymphoid	CD4+ T Cells, donor2 : CNhs11955 ctss	0,48	2,39	4,06	5,49	2,15	0,72	0	0,24	1,91
lymphoid	CD4+ T Cells, donor3 : CNhs11998 ctss	0,7	10,29	7,33	15,53	2,97	0,87	2,97	0,7	3,66
lymphoid	CD4+CD25+CD45RA+ naive regulatory T cells expanded, donor1 : CNhs13203 ctss	0	0,76	0,19	1,51	3,79	0	0,38	1,14	0,76
lymphoid	CD4+CD25+CD45RA+ naive regulatory T cells, donor1 : CNhs13238 ctss	0	2,78	3,62	12,25	2,78	0,56	4,18	0,56	5,01
lymphoid	CD4+CD25+CD45RA+ naive regulatory T cells, donor2 : CNhs13235 ctss	0	3,26	1,81	8,32	3,62	0	0,36	0,36	3,98
lymphoid	CD4+CD25+CD45RA+ naive regulatory T cells, donor3 : CNhs13513 ctss	1,66	1,66	3,33	11,65	1,66	0	0	1,66	11,65
lymphoid	CD4+CD25+CD45RA- memory regulatory T cells expanded, donor1 : CNhs13204 ctss	0	1,32	0,13	1,45	2,11	0,13	0,26	0,13	0,66
lymphoid	CD4+CD25+CD45RA- memory regulatory T cells expanded, donor2 : CNhs13811 ctss	0,14	1,25	0,28	1,67	0,7	0,14	0	0,7	0,56
lymphoid	CD4+CD25+CD45RA- memory regulatory T cells expanded, donor3 : CNhs13812 ctss	0	2,23	0,42	2,37	2,79	0,28	0,14	0,28	0,7
lymphoid	CD4+CD25+CD45RA- memory regulatory T cells, donor1 : CNhs13195 ctss	0,26	3,9	0,78	5,98	1,82	0,26	1,3	0,26	3,9
lymphoid	CD4+CD25+CD45RA- memory regulatory T cells, donor2 : CNhs13206 ctss	0,22	3,3	0,88	4,4	1,54	0,22	0	0,22	2,64
lymphoid	CD4+CD25+CD45RA- memory regulatory T cells, donor3 : CNhs13538 ctss	0	2,27	2,07	4,13	3,51	0	1,03	0,21	2,27
lymphoid	CD4+CD25-CD45RA+ naive conventional T cells expanded, donor1 : CNhs13202 ctss	0,32	0,95	0	1,91	0,64	0	0,64	0,64	0,32
lymphoid	CD4+CD25-CD45RA+ naive conventional T cells expanded, donor2 : CNhs13813 ctss	0	0,33	0	0,82	1,81	0	0	0,66	0,33
lymphoid	CD4+CD25-CD45RA+ naive conventional T cells expanded, donor3 : CNhs13814 ctss	0	0	0	1,71	1,99	0,28	0	0,85	0,28
lymphoid	CD4+CD25-CD45RA+ naive conventional T cells, donor1 : CNhs13223 ctss	0	1,57	2,8	9,06	3,24	0,11	3,47	0,78	6,82
lymphoid	CD4+CD25-CD45RA+ naive conventional T cells, donor2 : CNhs13205 ctss	0,63	1,47	0,42	4,82	3,56	0,21	0,42	0,84	3,35
lymphoid	CD4+CD25-CD45RA+ naive conventional T cells, donor3 : CNhs13512 ctss	0	5,56	0,93	9,27	8,34	0	1,85	0,93	10,2
lymphoid	CD4+CD25-CD45RA- memory conventional T cells expanded, donor1 : CNhs13215 ctss	0	0,26	0	1,83	1,44	0	0,26	0,39	0,26
lymphoid	CD4+CD25-CD45RA- memory conventional T cells, donor1 : CNhs13239 ctss	0	3,73	5,6	8,95	2,61	0,75	1,49	0,75	5,22
lymphoid	CD4+CD25-CD45RA- memory conventional T cells, donor2 : CNhs13237 ctss	0	3,58	2,98	11,34	0,9	0,6	0,9	0,3	4,77
lymphoid	CD4+CD25-CD45RA- memory conventional T cells, donor3 : CNhs13539 ctss	0,16	0,78	1,41	4,07	2,98	0,16	0,63	0,31	2,35
lymphoid	CD8+ T Cells (pluriselect), donor090309, donation1 : CNhs12176 ctss	0	1,19	0,8	5,97	3,58	0,4	0,4	0	1,19
lymphoid	CD8+ T Cells (pluriselect), donor090309, donation2 : CNhs12178 ctss	0	0	0	1,43	2,85	0	0	0,71	0,71
lymphoid	CD8+ T Cells (pluriselect), donor090309, donation3 : CNhs12180 ctss	0	1,07	0	2,14	0	0	0	1,07	0
lymphoid	CD8+ T Cells (pluriselect), donor090325, donation1 : CNhs12201 ctss	0,63	2,53	0,42	9,29	4,01	0,42	0,42	1,27	3,8
lymphoid	CD8+ T Cells (pluriselect), donor090325, donation2 : CNhs12199 ctss	0	0,49	0	2,92	3,57	0	0,32	0,81	0,65
lymphoid	CD8+ T Cells (pluriselect), donor090612, donation1 : CNhs12182 ctss	0	1,55	1,55	4,65	1,55	0	1,55	3,1	3,1
lymphoid	CD8+ T Cells (pluriselect), donor090612, donation2 : CNhs12184 ctss	0,57	1,41	0,28	2,83	3,11	0,57	0	0	1,41
lymphoid	CD8+ T Cells (pluriselect), donor090612, donation3 : CNhs12187 ctss	0	3,04	0	4,06	2,03	0	0	0	1,01
lymphoid	CD8+ T Cells, donor1 : CNhs10854 ctss	0	5,41	5,02	11,01	2,51	0,19	0,77	0,77	3,48
lymphoid	CD8+ T Cells, donor2 : CNhs11956 ctss	0,39	7,89	5,72	6,31	0,59	1,18	0,39	0,2	2,76
lymphoid	CD8+ T Cells, donor3 : CNhs11999 ctss	0,39	5,27	4,24	10,15	3,08	0,77	1,8	0,51	1,93
myeloid_low	Basophils, donor1 : CNhs12546 ctss	2,37	26,11	2,71	33,23	4,07	1,02	2,37	2,03	3,73
myeloid_low	Basophils, donor2 : CNhs12563 ctss	1,17	13,64	2,73	26,12	3,51	0,39	1,56	3,9	5,85
myeloid_low	Basophils, donor3 : CNhs12575 ctss	13,67	16,61	3,68	53,66	5,73	2,65	1,62	1,76	8,82
myeloid_low	Macrophage - monocyte derived, donor1 : CNhs10861 ctss	0	0	0,24	3,13	3,85	0	0	0,24	0
myeloid_low	Macrophage - monocyte derived, donor2 : CNhs11899 ctss	0,94	0,23	0	0,94	8,67	0,7	0,94	0,7	0,47
myeloid_low	Macrophage - monocyte derived, donor3 : CNhs12003 ctss	0,43	0,14	0,28	3,27	6,69	0,43	0,14	0,85	0,43
myeloid_low	Mast cell, donor1 : CNhs12566 ctss	0	0,21	0,41	1,14	2,07	0,21	0	0,72	0,1
myeloid_low	Mast cell, donor2 : CNhs12594 ctss	0,77	12,54	0,77	16,77	4,48	0	1,28	1,28	3,33
myeloid_low	Mast cell, donor3 : CNhs12593 ctss	0,12	7,94	0,98	15,87	5,98	0,98	0,61	0,85	2,56
myeloid_low	Mast cell, donor4 : CNhs12592 ctss	0,36	7,82	1,07	28,57	5,1	0,95	0,95	2,25	6,16
CNS	occipital lobe, fetal, donor1 : CNhs11784 ctss	0,35	0	0,18	1,23	7,37	0	0,18	5,97	0,18
CNS	parietal lobe, fetal, donor1 : CNhs11782 ctss	0	0,32	0	0,97	2,91	0,32	0,97	11,31	0
CNS	temporal lobe, fetal, donor1, tech rep1 : CNhs11772 ctss	0	0	0	0,65	6,46	0	0,32	2,59	0
CNS	temporal lobe, fetal, donor1, tech rep2 : CNhs12996 ctss	0	0	0	1,51	2,26	0	0,25	0,5	0

Suppl. Table 2 Expression of *C9orf72* TSSs as defined in CAGEseq dataset 2

mutationGroup	donorGroup	regionGroup	S4-minus	AS1-plus	S2C-minus	S2B-minus	S2A-minus	AS2-plus	S1+S3a-minus	S1+S3b-minus	AS3-plus
FTD-C9orf72	02218	caudate	0,11	0,37	0,07	0,85	2,44	0	0,04	5,1	0,15
FTD-C9orf72	02218	cerebellum	0,45	0,26	0,08	2,07	10,02	0,23	1,39	7,99	1,17
FTD-C9orf72	02218	frontal	0,04	0,85	0,04	0,7	6,68	0	0,5	5,68	0,27
FTD-C9orf72	02218	occipital	0,12	0,12	0	1,11	9,9	0	0	3,59	0,25
FTD-C9orf72	02218	putamen	0	1,59	0,06	0,34	3,91	0	0,34	3,23	0,06
FTD-C9orf72	02218	temporal	0,12	0,48	0	1,21	4,6	0	0,24	4,48	0
FTD-C9orf72	09218	caudate	0,1	1,08	0,1	1,49	3,04	0,15	0,21	5,4	1,44
FTD-C9orf72	09218	cerebellum	0,63	0,34	0	3,44	9,06	0,29	0,69	6,25	1,32
FTD-C9orf72	09218	frontal	0,06	0,36	0,12	1,42	2,78	0,47	0,3	4,44	0,53
FTD-C9orf72	09218	occipital	0	0,87	0,19	1,54	3,56	0,29	0,1	4,82	0,19
FTD-C9orf72	09218	putamen	0,25	1,43	0,2	1,13	2,66	0,2	0,15	4,48	0,25
FTD-C9orf72	09218	temporal	0	0,62	0	1,85	4,71	0,31	0,08	5,25	0,62
FTD-C9orf72	10166	caudate	0,28	0,28	0,14	2,93	8,15	0,07	0,21	2,58	1,18
FTD-C9orf72	10166	cerebellum	0	0,04	0	13,76	25,1	0,36	0,28	6,44	0,52
FTD-C9orf72	10166	frontal	0,17	0	0,06	2,07	19,63	0,17	0,29	4,76	0,34
FTD-C9orf72	10166	hippo	0,36	0	0,09	2,18	13,92	0,18	0,09	3,09	0,18
FTD-C9orf72	10166	occipital	0,79	0	0	2,65	21,81	0	0,26	5,92	0,09
FTD-C9orf72	10166	temporal	0,16	0,16	0,24	3,19	16,53	0,48	0,08	4,23	0,32
FTD-C9orf72	97231	caudate	0	0,96	0	0,13	3,02	0	0	3,66	0,19
FTD-C9orf72	97231	cerebellum	0,04	0,16	0	5,29	9,4	0,16	0,2	4,15	0,51
FTD-C9orf72	97231	frontal	0,23	0,47	0,1	0,78	3,95	0,03	0,34	5,35	0,99
FTD-C9orf72	97231	hippo	0,07	0,66	0,03	1,18	7,63	0,07	0,35	6,69	0,45
FTD-C9orf72	97231	occipital	0,11	2,42	0,03	0,68	4,66	0,16	0,16	4,49	0,71
FTD-C9orf72	97231	putamen	0	0,29	0	0,18	2,11	0,18	0	3,29	0,12
FTD-C9orf72	97231	temporal	0,17	1,08	0,22	0,91	6,66	0,09	0,35	4,84	0,26
FTD-C9orf72	97303	caudate	0,26	0,73	0	0,34	1,55	0	0,09	3,36	0,3
FTD-C9orf72	97303	cerebellum	0,39	0	0	3,24	9,43	0	0,53	8,07	0,48
FTD-C9orf72	97303	frontal	0,1	0,86	0	0,67	7,23	0	0,29	4,85	0,38
FTD-C9orf72	97303	hippo	0,15	0,25	0,05	0,81	3,58	0	0,25	6,66	0,81
FTD-C9orf72	97303	occipital	0	2,07	0	0,59	9,93	0	0,69	9,24	0,89
FTD-C9orf72	97303	putamen	0,19	0,39	0	0,31	3,37	0	0,39	5,86	0,35
FTD-C9orf72	97303	temporal	0	0,97	0	1,12	7,97	0	0,67	8,64	0,37
FTD-GRN	07083	caudate	0,35	0,06	0,12	1,75	4,21	0,12	0	2,46	0,64
FTD-GRN	07083	frontal	0,29	0	0,06	2,84	14,36	0,26	0,03	5,33	0,61
FTD-GRN	07083	hippo	0,15	0,15	0	2,42	5,53	0,49	0,1	3,65	0,1
FTD-GRN	07083	putamen	0,76	0	0,23	1,29	6,08	0,15	0	3,27	1,37
FTD-GRN	07083	temporal	0,91	0	0	2,33	10,97	0,4	0,11	3,75	0,28
FTD-GRN	07106	caudate	1,53	0,26	0	7,02	5,23	0	0,26	11,36	3,45
FTD-GRN	07106	cerebellum	0,22	0	0	5,95	9,25	0,22	0,95	8,66	0,51
FTD-GRN	07106	frontal	0,23	0	0	2,5	3,87	0	0,06	8,54	0,63
FTD-GRN	07106	hippo	0,34	0,08	0	2,79	2,79	0	0,25	6,86	1,86
FTD-GRN	07106	occipital	0,5	0,23	0,08	1,65	4,5	0,15	0,15	6,61	1,38
FTD-GRN	07106	putamen	0,08	0	0	1,91	2,9	0	0	8,02	0
FTD-GRN	07106	temporal	0,12	0	0,08	2,09	5,03	0,08	0	5,36	0,53
FTD-GRN	08244	caudate	0	0	0,06	1,06	2,39	0,11	0,72	8,79	0,17
FTD-GRN	08244	cerebellum	1,6	0,06	0	3,94	10,84	0	0,62	14,23	5,42
FTD-GRN	08244	frontal	0,1	0	0	0,49	3,53	0	0,2	3,92	0,69
FTD-GRN	08244	occipital	0,3	0	0	0,59	3,12	0	0	8,48	0,15
FTD-GRN	08244	putamen	0	0	0	0,13	1,25	0	0,38	4,01	0
FTD-GRN	08244	temporal	0,17	0	0,11	0,5	2,23	0,06	0,34	4,8	0,28
FTD-GRN	09126	caudate	0,46	0,2	0,13	4,32	4,03	0	0,1	6,7	0,07
FTD-GRN	09126	cerebellum	2,73	0	0	4,8	12,99	0,94	0	7,81	6,31
FTD-GRN	09126	frontal	0,26	0	0	2,97	3,62	0,26	0	2,33	0,39
FTD-GRN	09126	hippo	0,08	0,16	0	1,55	6,37	0	0,08	1,55	0,93
FTD-GRN	09126	occipital	0,51	0	0,25	1,27	8,6	0,25	0	2,15	1,52
FTD-GRN	09126	putamen	0,24	0	0,16	2,97	4,42	0,56	0,16	3,86	0,08
FTD-GRN	09126	temporal	0,34	0	0	2,31	3,94	0,43	0	2,48	0,77
FTD-GRN	10200	caudate	0,2	0	0,23	4,07	5,09	0	0,27	7,64	1,29
FTD-GRN	10200	cerebellum	1,83	0,13	0	15,11	13,1	1,01	1,26	12,28	2,39
FTD-GRN	10200	frontal	0,39	0	0,09	3,01	5,59	0,13	0,35	5,59	0,7
FTD-GRN	10200	hippo	0,08	0	0,16	3,32	6,25	0	0	6,88	0,71
FTD-GRN	10200	occipital	0,57	0	0	2,5	6,07	0,12	0	5,54	0,49
FTD-GRN	10200	putamen	0	0,2	0	2,62	5,05	0	0	1,62	0,2
FTD-GRN	10200	temporal	0,29	0	0,04	2,34	5,99	0,12	0,57	4,56	1,19
FTD-MAPT	00136	caudate	0,28	0,04	0,2	3,66	4,96	0,24	0,49	7,11	7,15
FTD-MAPT	00136	cerebellum	0,21	0,05	0	7,65	12,65	0,47	0,16	7,34	0,26
FTD-MAPT	00136	frontal	0,23	0	0	1,28	3,55	0	0,29	6,8	0,41
FTD-MAPT	00136	hippoR	0	0	0,15	1,57	5,76	0	0,3	7,03	1,05
FTD-MAPT	00136	occipital	0,35	0	0	1,21	5,54	0	0,52	6,93	0,69
FTD-MAPT	00136	putamen	0,14	0	0	0,82	2,82	0,09	0,45	6,64	0,68
FTD-MAPT	00136	temporal	0	0	0	0,87	2,91	0,15	0,58	5,67	0,87
FTD-MAPT	04245	caudate	0,12	0,06	0,18	3,44	3,74	0	0,55	8,04	2,33

FTD-MAPT	04245 cerebellum	0,33	0	0	6,52	18,56	0,17	1,17	9,44	0,67
FTD-MAPT	04245 frontal	0,24	0,24	0	2,82	4,21	0	0,36	13,16	4,09
FTD-MAPT	04245 hippo	0,14	0	0	1,27	3,67	0	0,14	5,44	0,49
FTD-MAPT	04245 occipital	0,12	0,23	0	0,93	7,43	0	0,58	8,25	0
FTD-MAPT	04245 putamen	0	0	0,33	0,67	4,88	0	0,55	7,1	0,44
FTD-MAPT	04245 temporal	0,31	0	0,24	1,14	6,75	0,07	0,76	8,51	1
FTD-MAPT	05150 cerebellum	0,67	0	0,06	6,11	20,65	0,22	1,89	8,66	2,61
FTD-MAPT	05150 frontal	0,08	0	0,08	1,01	12,36	0,25	0,52	5,55	0,82
FTD-MAPT	05150 occipital	0,41	0	0,12	0,46	15,24	0,06	0,41	8,63	1,33
FTD-MAPT	05150 temporal	0,14	0,04	0,07	1,01	12,72	0	0,54	4,59	0,47
FTD-MAPT	09070 caudate	0,34	0	0,11	3,03	8,06	0,11	0,17	2,63	1,83
FTD-MAPT	09070 cerebellum	1,1	0	0	11,41	19,51	0,42	1	7,73	1,21
FTD-MAPT	09070 frontal	0,14	0,56	0	1,99	4,26	0,05	0,37	1,48	0,69
FTD-MAPT	09070 hippo	0,25	0	0	2,34	4,35	0,17	0,08	3,01	0,59
FTD-MAPT	09070 occipital	0,51	0	0	1,73	9,87	0,31	0,1	4,37	1,63
FTD-MAPT	09070 putamen	0	0	0,12	0,77	6,63	0,16	0,33	2,28	0,33
FTD-MAPT	09070 temporal	0	0,15	0	1,62	5,59	0,51	0,22	1,47	1,03
FTD-MAPT	92017 cerebellum	0	0	0,05	0,49	21,6	0,05	0,27	6,4	0,49
FTD-MAPT	92017 frontal	0,1	0	0	1,14	10,76	0	0,19	3,52	0,33
FTD-MAPT	92017 hippo	0,03	0	0,03	0,67	5,77	0	0,09	2,73	0,43
FTD-MAPT	94198 caudate	0,07	0,07	0,37	1,03	2,35	0,73	0,29	2,49	1,25
FTD-MAPT	94198 putamen	0,22	0	0,07	1,62	1,54	1,32	0,07	1,54	0,22
FTD-MAPT	96328 temporal	0	0,14	0	0,55	1,24	0,41	0	0,69	0,28
Control	05217 caudate	0,16	0,08	0,16	1,52	10,06	0,24	0,4	1,6	0,56
Control	05217 cerebellum	0,34	0	0,05	6,15	22,57	0,78	0,44	4,55	0,58
Control	05217 frontal	0,08	0	0	1,16	21,25	0,08	0,91	4,47	0,17
Control	05217 hippo	0	0	0	1,95	9,77	0	0,16	1,14	0,16
Control	05217 occipital	0	0	0	0,59	8,87	0,2	0	2,17	0,2
Control	05217 putamen	0	0,1	0	0,89	8,16	0,1	0,69	1,28	0,3
Control	05217 temporal	0,09	0	0	1,56	16,26	0,26	0,43	4,41	0
Control	10196 caudate	0,04	0,38	0	1,62	10,27	0,17	0,81	10,22	0,6
Control	10196 cerebellum	0,71	0	0,07	5,26	26,53	0,21	2,2	17,85	0,71
Control	10196 frontal	0,14	0,03	0,07	1,15	14,05	0	0,84	14,75	0,17
Control	10196 hippo	0,11	0	0	1,22	10,39	0	0,11	11,16	0
Control	10196 occipital	0,13	0	0,04	1,56	8,53	0	0,4	13,4	0,4
Control	10196 putamen	0	0	0,1	1,58	13,96	0	2,82	10,1	0,2
Control	10196 temporal	0,27	0	0,07	0,81	12,65	0	1,01	13,47	0,34
Control	10258 caudate	0,12	0,16	0,16	1,15	10,39	0,08	1,35	7,97	0,44
Control	10258 cerebellum	0,3	0,15	0,15	5,44	35,31	0,22	2,76	14,53	0,74
Control	10258 frontal	0	0	0,23	0,78	15,67	0,08	0,93	11,02	0,31
Control	10258 hippo	0,24	0	0,08	1,98	13,01	0	0,48	8,57	0,48
Control	10258 occipital	0,23	0	0	0,7	19,25	0	0,6	11,32	0,8
Control	10258 putamen	0,12	0	0,17	0,87	7,88	0,06	0,58	7,99	0,17
Control	10258 temporal	0,27	0,05	0,11	1,07	14,57	0	0,97	9,57	0,21
Control	10327 caudate	0,08	0	0,21	2,7	9,71	0,08	0,95	7,64	0,42
Control	10327 cerebellum	1,14	0,03	0	3,81	19,2	0,31	1,38	14,32	2,01
Control	10327 frontal	0,14	0,05	0,05	1,82	15,22	0,05	0,68	14,76	0,5
Control	10327 hippo	0,19	0,06	0	2,5	10,2	0	0,31	8,57	0,56
Control	10327 occipital	0,33	0	0,13	1,33	15,04	0,07	0,6	10,93	0,99
Control	10327 putamen	0,18	0	0	1,36	6,95	0,04	0,48	6,58	0,26
Control	10327 temporal	0,12	0,2	0,08	1,83	16,91	0	1,26	12,26	0,24
Control	11040 caudate	0,18	0,13	0,05	1,23	7,69	0	1,05	6,79	0,53
Control	11040 cerebellum	2,13	0	0,19	4,45	17,82	0,19	2,13	16,65	1,55
Control	11040 frontal	0,19	0	0,03	1,4	15,43	0	0,42	12,09	0,13
Control	11040 hippo	0	0	0	1,68	7,92	0,05	0,27	7,38	0,27
Control	11040 occipital	0,16	0	0,16	0,88	19,79	0	0,4	10,53	0,16
Control	11040 putamen	0,11	0,07	0,04	1,52	8,76	0	1,41	10,36	0,37
Control	11040 temporal	0,06	0,06	0,11	1,29	11,55	0	0,84	8,86	0,34
Control	12005 caudate	0,24	0	0,07	1,23	10,75	0	0,86	10,89	0,27
Control	12005 cerebellum	0,63	0	0	3,96	29,21	0,21	3,34	14,5	0,42
Control	12005 frontal	0,18	0,05	0,1	1,15	16,17	0	0,94	15,05	0,31
Control	12005 hippo	0,15	0	0	1,16	13,83	0	1,24	10,52	0,49
Control	12005 occipital	0,38	0	0,05	1,29	19,41	0	0,38	15,79	0
Control	12005 putamen	0,15	0,03	0,23	1,03	12,41	0	2,11	9,33	0,31
Control	12005 temporal	0,26	0	0,04	1,91	17,16	0	1,43	13,3	0,39
Control	12062 caudate	0,18	0	0,09	0,72	15,06	0	0,18	3,14	0
Control	12062 cerebellum	0,52	0	0,18	4,12	38,76	0,15	1,31	14,01	0,55
Control	12062 frontal	0	0	0,08	1,08	24,56	0,08	0,5	7,99	0,08
Control	12062 hippo	0,34	0,07	0	0,88	17,23	0	0,27	6,46	0,27
Control	12062 occipital	0,09	0	0,18	1,31	28,79	0	0,26	8,49	0,26
Control	12062 putamen	0,08	0,04	0,04	1,09	12,54	0	0,35	5,88	0
Control	12062 temporal	0,05	0,05	0	0,91	20,24	0	0,71	6,41	0,4

Suppl. Table 3 List of primers used in this study

Primers Name	Sequence	Assay
AS1-F	GCATTGCTGCCCTCATATGC	qPCR
AS1-R	ATGAGATGGGGGTGTGGAGA	
AS2-F	CGACTCCTGAGTTCCAGAGC	qPCR
AS2-R	CACAGTACTCGCTGAGGGTG	
AS3-F	TGCATTCAAAACTCCACTGC	qPCR
AS3-R	AAAGGCAAAATTGCGATGAC	
TSS4-F	TGGGAGAGTAGTTGCCTGGT	qPCR
TSS4-R	CGACATCACTGCATTCCAAC	
TSS4-F	TGCTCATTGGGTCTATCTGG	RT-PCR
TSS4-X5utr-R	AACACAAATTTAAGCAACAGTTCAA	
TSS4-X11utr-R	TGAGCTACTTTACCAGCGATCA	
LK-ssRT-AS1	CGACTGGAGCACGAGGACACTGAGGTTCGCTAGGAACCCGAGACGGTC	ssRT
AS1F-short	GGTTCGCTAGGAACCCGAGACGGTC	ssRT-PCR
ssRT-AS1R	GTGAAGCAACCAGGTCATGTCCCAC	ssRT-PCR
LK-ssRT-AS2	CGACTGGAGCACGAGGACACTGAACGTAACCTACGGTGTCCCGCTAGGA	ssRT
AS2F-short	ACGTAACCTACGGTGTCCCGCTAGGA	ssRT-PCR
ssRT-AS2R	CGACTCCTGAGTTCCAGAGCTTGCT	ssRT-PCR
LK-ssRT-AS3	CGACTGGAGCACGAGGACACTGACAGTGTGAAAATCATGCTTGAGAGAA	ssRT
AS3F- short	CAGTGTGAAAATCATGCTTGAGAGAA	ssRT-PCR
ssRT-AS3R	CTGGAGAAAGTGAAGACGATTTCGTG	ss-RT-PCR
LK	CGACTGGAGCACGAGGACACTGA	ss-RT-PCR
AS1-GS1	CCATCTCATCCCGCATGATCTCCTC	3'-RACE
AS1-GS2	CTAGCGAACCCCGACTTGGTC	3'-RACE
AS2-GS1	GAGTTCCAGAGCTTGCTACAGGCTG	3'-RACE
AS2-GS2	GTTCACCCTCAGCGAGTACTGTG	3'-RACE
AS3-GS1	GGAGCGTTGGCGCAATAGCGTGTG	3'-RACE
AS3-GS2	TGCTGGATCTGGAGAAAGTGAAGACGA	3'-RACE

gC9orf72-x1aF	TGCAGTGGAGTTTTGAATGC	1.6kB PCR
gC9orf72-x1aR	TCCTGAGTTCCAGAGCTTGC	
gC9orf72-x1aF1	CAGGGTTTGCAGTGGAGTTT	1.6kB PCR
gC9orf72-x1aR1	CTACAGGCTGCGGTTGTTTC	
gC9orf72-x1bF	TGCGGTTGCGGTGCCTGCGCCC	1.6kB PCR
gC9orf72-x1bR	AGCGGCAGCGCTCCCAGCGGGT	
gC9-UTR1F	TCAGTGTAGAAACTTGGTAAATGC	1.6kB PCR
gC9-UTR1R	CATTCACCGAGGAAGAAAGG	
gC9-UTR2F	AAGGATCTGAGGAGCTGGTG	1.6kB PCR
gC9-UTR2R	GGTCTGCAGGTGTATAGATTCC	
gC9-UTR3F	CCATCTCACACTTGCAGATCA	1.6kB PCR
gC9-UTR3R	GAAATGGGGCGGGGCAAC	
gC9-UTR4F	AGAGCAGGTGTGGGTTTAGG	1.6kB PCR
gC9-UTR4R	GCAATTCCACCAGTCGCTAG	

Suppl. Table 4 Biological functions significant to the three modules related to *C9orf72* TSSs as identified by WGCNA

Modules with *C9orf72* TSSs	Functions	p-Value
Yellow_S2A TSS	Neurotransmission	1.34E-55
	Synaptic transmission	1.16E-42
	Long term potentiation	6.68E-23
	Potentiation of synapse	3.31E-22
	Action potential of neurons	2.82E-18
Turquoise_S1+S3b TSS	Microtubule dynamics	4.44E-34
	Organization of cytoskeleton	1.40E-31
	Organization of cellular protrusion	8.10E-31
	Organization of cytoplasm	9.66E-31
	Neuritogenesis	1.35E-22
Blue_all remaining TSSs	Leukocytes migration	6.16E-85
	Chemotaxis of phagocytes	8.58E-44
	Chemotaxis of myeloid cells	1.02E-42
	Migration of phagocytes	1.38E-38
	Recruitment of phagocytes	2.67E-37

The WGCNA yielded 18 co-expression modules ranging in size from 29 to 15.731 TSSs. The *C9orf72* TSSs with distinct mode of expression in CNS and myeloid cells segregated into three distinct co-expression modules that we named yellow, turquoise and blue. The S1b+S3b and S2A TSSs, that show comparable expression level in CNS and white blood cells, fell into the turquoise and yellow modules respectively, while all the remaining TSSs, characterized by higher expression in myeloid_high cells, fell into the blue module.

We performed functional enrichment analysis using IPA to highlight biological processes and pathways enriched in the three modules. The top 5 significant biological functions are here reported.

Suppl. Table 5 Biological functions significant for genes that correlate with *C9orf72* TSSs in the WGCNA identified modules

C9orf72 TSSs	Functions	p-Value
S2A_correlations	Transport of vescicles	7.17E-0.6
	Neurotransmission	1.56E-05
	Neuritogenesis	5.02E-05
	Organization of synapse	3.49E-04
S1+S3a_correlations	Chemotaxis of leukocytes	4.12E-09
	Organization of cytoskeleton	1.04E-08
	Organization of cellular protrusion	1.22E-06
AS3_correlations	Inflammatory response	8.38E-68
	Cell movement of phagocytes	8.28E-59
	Immune response of cells	4E-56

The table shows some among the most significant expression correlations between *C9orf72* S2A TSS and genes in the yellow module and between *C9orf72* S1+S3a TSS and *C9orf72* AS3 TSS with genes in the blue module.

Suppl. Table 6 Summary of the Mann-Whitney test performed on NRQ values from qPCR experiments on medial frontal gyrus

Target	Disease group	FRD adjusted value
C9orf72 total	*C9orf72*-HRE	0,008620717
	FTD-*GRN*	0,008620717
	FTD-*MAPT*	0,013840534
C9orf72 transcript 1	*C9orf72*-HRE	0,010777048
	FTD-*GRN*	0,013533
	FTD-*MAPT*	0,013840534
C9orf72 transcript 2	*C9orf72*-HRE	0,010777048
	FTD-*GRN*	0,219105706
	FTD-*MAPT*	0,097343429
C9orf72 transcript 3	*C9orf72*-HRE	0,005388524
	FTD-*GRN*	0,005747145
	FTD-*MAPT*	0,005388524
C9orf72-AS1	*C9orf72*-HRE	0,23059757
	FTD-*GRN*	1
	FTD-*MAPT*	1
C9orf72-AS2	*C9orf72*-HRE	0,716652933
	FTD-*GRN*	0,716652933
	FTD-*MAPT*	1
C9orf72-AS3	*C9orf72*-HRE	0,010777048
	FTD-*GRN*	0,03143996
	FTD-*MAPT*	0,08144598

Results from the Mann-Whitney tests performed using NRQ values from qPCR experiments in medial frontal gyrus using *C9orf72*-HRE (N=10); FTD-*GRN* mutation carriers (N=8) and FTD-*MAPT* mutation carriers (N=13) as compared to control donors (N=5).
